# Src-NADH dehydrogenase subunit 2 complex and recognition memory of imprinting in domestic chicks

**DOI:** 10.1371/journal.pone.0297166

**Published:** 2024-01-29

**Authors:** Lela Chitadze, Maia Meparishvili, Vincenzo Lagani, Zaza Khuchua, Brian J. McCabe, Revaz Solomonia

**Affiliations:** 1 Institute of Chemical Biology, School of Natural Sciences and Medicine, Ilia State University, Tbilisi, Georgia; 2 Biological and Environmental Sciences and Engineering Division, King Abdullah University of Science and Technology, Thuwal, Saudi Arabia; 3 Department of Zoology, University of Cambridge, Cambridge, United Kingdom; 4 Iv. Beritashvili Centre of Experimental Biomedicine, Tbilisi, Georgia; Showa University School of Dentistry, JAPAN

## Abstract

Src is a non-receptor tyrosine kinase participating in a range of neuronal processes, including synaptic plasticity. We have recently shown that the amounts of total Src and its two phosphorylated forms, at tyrosine-416 (activated) and tyrosine-527 (inhibited), undergoes time-dependent, region-specific learning-related changes in the domestic chick forebrain after visual imprinting. These changes occur in the intermediate medial mesopallium (IMM), a site of memory formation for visual imprinting, but not the posterior pole of the nidopallium (PPN), a control brain region not involved in imprinting. Src interacts with mitochondrial genome-coded NADH dehydrogenase subunit 2 (NADH2), a component of mitochondrial respiratory complex I. This interaction occurs at brain excitatory synapses bearing NMDA glutamate receptors. The involvement of Src-NADH2 complexes in learning and memory is not yet explored. We show for the first time that, independently of changes in total Src or total NADH2, NADH2 bound to Src immunoprecipitated from the P2 plasma membrane-mitochondrial fraction: (i) is increased in a learning-related manner in the left IMM 1 h after the end of training; (ii), is decreased in the right IMM in a learning-related way 24 h after training. These changes occurred in the IMM but not the PPN. They are attributable to learning occurring during training rather than a predisposition to learn. Learning-related changes in Src-bound NADH2 are thus time- and region-dependent.

## Introduction

Src is a non-receptor tyrosine kinase playing key roles in developmental, physiological and pathological processes [1]. Src is also involved in neurotransmitter receptor function, neurotransmitter release and synaptic plasticity [2]. Phosphorylation of Src at tyrosine-527 (527P-Src) is effected by other kinases and inhibits Src activity, whereas autophosphorylation of tyrosine-416 (416P-Src), in Src’s activation loop, increases Src activity (2). Several phosphorylation substrates have been identified for activated Src [3]. Src interacts with mitochondrially-encoded NADH dehydrogenase subunit 2 (NADH2) at a region of low sequence conservation in Src termed the unique domain [4]. This interaction occurs outside mitochondria at excitatory synapses in the brain and also involves the N-methyl-D-aspartate (NMDA) receptor complex [4, 5].

We have shown previously that, 1 h and 24 h after the end of visual imprinting in domestic chicks, there are complex learning-related changes in the total amount of Src and its two phosphorylated forms (416P-Src and 527P-Src) in the intermediate medial mesopallium (IMM), a site of memory encoding in the chick forebrain [6]. The results indicate that there are two pools of Src; one in which active Src influences a predisposition to learn well, and the second in which Src is inhibited and which occurs only when learning occurs [6]. By ‘predisposition’ is meant a quality that is present before imprinting training. In that study, Src/NADH2 interaction was not investigated and, as far as we know, the role of this interaction in learning and memory has not yet been established.

Visual imprinting in the chick entails learning about a specific visual stimulus. When exposed to the stimulus (training), a chick learns about it and demonstrates this learning by expressing a social preference for the stimulus [7–9]. Visual imprinting possesses several favorable characteristics for memory research. (i) A brain region has been identified as a site of memory encoding for the training stimulus. This region is the intermediate medial mesopallium (IMM), formerly known as the IMHV [10]. (ii) Memory strength may be measured in terms of behavioral preference for the training stimulus–a preference score, which expresses approach to the training stimulus relative to approach to a stimulus not previously seen. One may then determine, by linear modelling, whether molecular events in the IMM (and in other brain regions known not to be involved in imprinting) are related to a behavioural measure of the strength of learning (i.e. preference score) or to measures that are not associated with learning or memory. (iii) Variance that is unexplained by covariation with learning (i.e. residual variance from a regression with preference score) can enable discrimination between a *result* of learning or an influence on learning ability established before training occurs—a *predisposition to learn*; see e.g. [11]. (iv) Imprinting can occur with minimal sensory experience before training, giving a minimal baseline against which learning-related neural changes may relatively easily be detected. Criteria for inferring that a change is learning-related and the basis for interpreting effects on residual variance have been published [11–14]. Using these criteria we have demonstrated a progression of learning-related molecular changes in the IMM over the 24 h after training [6, 11, 15–29].

A common feature of neural changes underlying visual imprinting in chicks is hemispheric asymmetry. Lesion experiments indicate that the left and the right IMM have storage functions but that, in addition, the right IMM is necessary for storage outside the IMM in a region termed S’ [9, 13, 30, 31]. The location of S’ is not yet known but possibilities have been proposed. Changes in the amount of neural cell adhesion molecule 180 (NCAM 180) implicate the hyperpallium apicale (HA) [27]. c-fos mapping data suggest several brain regions which may represent the S’ system [32]. Functional MRI studies indicate that the network within the “prefrontal” nidopallium caudolaterale (NCL) could be a central hub for S’ [33].

After training, learning-related molecular changes occur on both sides of the IMM but during the first day after training, there is a trend towards lateralization, the left side of this structure predominating [14].

Most significant correlations between molecular changes and preference score are attributable to learning during training. However, we have identified molecular species whose levels are significantly correlated with preference score, but which reflect a predisposition to learn rather than being a consequence of learning. These species are: (i) the micro-RNA gga-miR-130b-3p (negative correlation), (ii) 416P-Src (positive correlation) and (iii) aggregated form of cytoplasmic polyadenylation element binding protein 3 (CPEB3-AF) in the left IMM (positive correlation) [6, 11, 21]; see also Discussion.

In the present study we show for the first time that, independently of changes in total Src or total NADH2, NADH2 bound to Src immunoprecipitated from the P2 plasma membrane-mitochondrial fraction: (i) is increased in a learning-related manner in the left IMM 1 h after the end of training; (ii) is decreased in the right IMM in a learning-related way 24 h after the end of training. These changes are thus time- and region-dependent. The evidence indicates that they are attributable to learning that occurred during training and not to a predisposition to learn.

## Results

### Behavior

Mean preference scores in the 1 h and 24 h groups were 76.7 ± 3.4 SEM and 76.2 ± 4.7 SEM respectively. At both times, mean preference score significantly exceeded the ‘no preference’ score of 50 (*t*-test, *P* < 0.0001). Mean approach during training and testing was 215.0 ± 48.0 and 68.0 ± 20.0 meters respectively for the 1 h group, and 232.0 ± 46.0 and 69.6 ± 14.0 meters respectively for the 24 h group. Approach during training and testing did not differ significantly between the two times after training.

### Immunostaining

Antibodies against NADH2 reacted with a protein of molecular weight 38 kDa, whereas antibodies against Src reacted with a protein band of 60 kDa (S1 Fig). In both cases molecular weights of stained bands correspond to those of NADH2 and Src respectively (https://www.uniprot.org/uniprotkb/P00523/entry and https://www.uniprot.org/uniprotkb/P18937/entry). The specificity of NADH2 and Src staining was confirmed with blocking peptides (S2 Fig). For both Src and NADH2, plotting of optical densities of internal standards against amount of protein (15, 30, 45 and 60 μg protein of P2 fraction) showed a virtually perfect fit to a straight line (S1 Fig).

### IA chromatography

#### No Src or NADH2 was retained on Control Agarose resin

To check if Src or NADH2 was retained on Control Agarose resin during pre-clearance of the RIPA-soluble fraction, this fraction was applied to Control Agarose resin columns and elution carried out as for the Src antibody cross-linked columns (see below). No Src or NADH2 immunoreactivity was detected in the eluates, indicating that neither protein had been bound to the Control Agarose resin.

#### Src protein was completely adsorbed during IA chromatography

No Src immunoreactivity was detected in the flow-through of Src antibody affinity columns, indicating complete binding of Src protein to the columns.

#### IA chromatography quantitatively reflected levels of Src and Src-NADH2 complexes

To confirm that our conditions of IA chromatography permitted accurate measurement of Src and Src-NADH2 complexes, four different amounts (50, 100, 150 and 200 μg of protein) of RIPA buffer-solubilized and Protein A/G agarose-precleared fractions were applied to Src antibodies immobilized on columns. Western blots of eluates were stained with antibodies against Src and NADH2 proteins. For both proteins, the optical densities of immunostained bands were plotted against amounts of corresponding protein. For both Src and NADH2, least-squares regression showed an excellent fit to a straight line (S3 Fig).

#### NADH2 interacted with both phosphorylated forms of Src

We carried out immunoaffinity chromatography on antibodies against NADH2 and affinity eluates were immunostained for 416P-Src and 527P-Src. Both active (416P-Src) and inhibited (527P-Src) forms of Src bound to NADH2 (S4 Fig).

### Changes in amount of Src, NADH2 and Src-NADH2 complexes

**1 h after the end of training**.

### Left IMM

#### NADH2-IP

**Fig 1:**
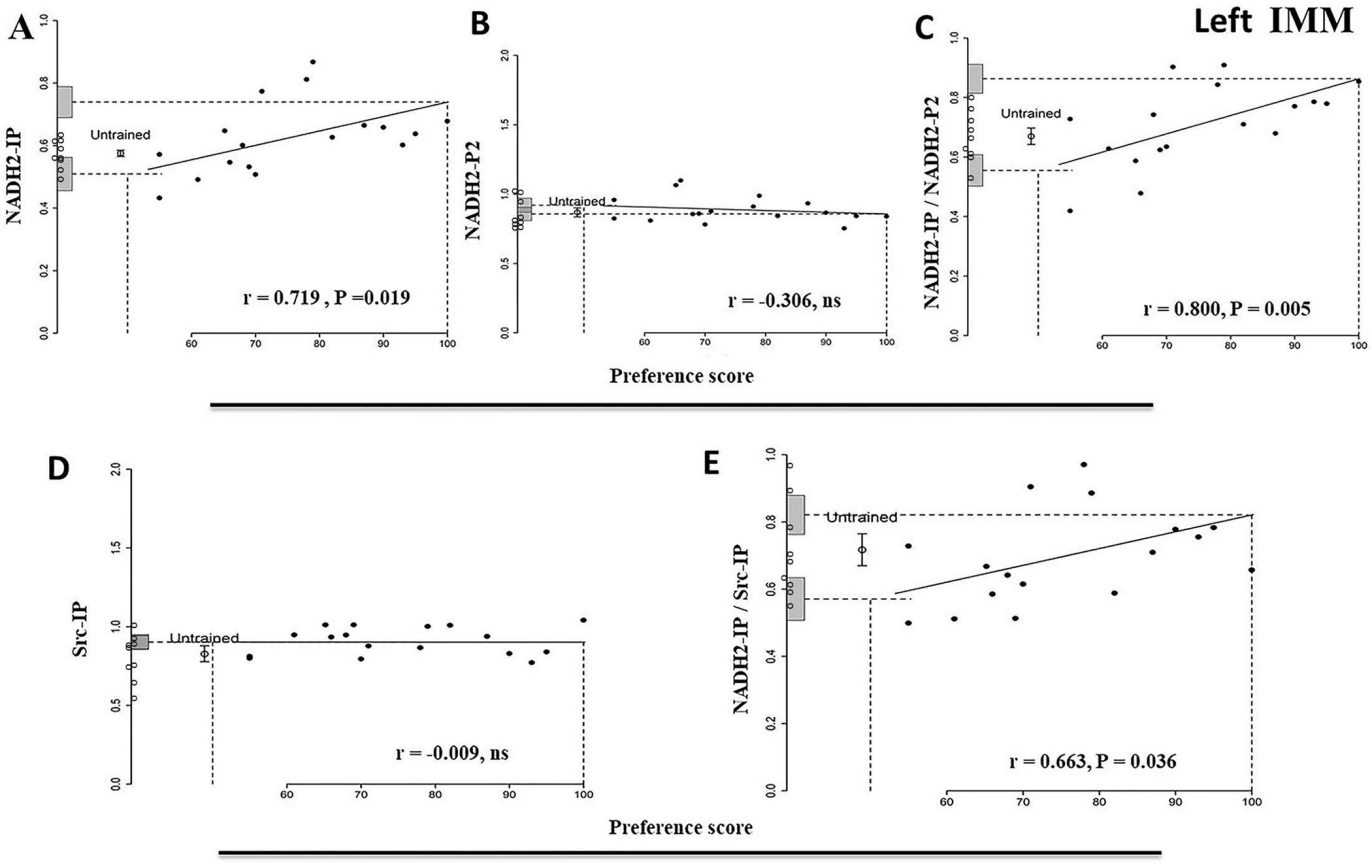
Left IMM 1 h after the end of training. Preference score plotted against standardized relative amounts of NADH2-IP (A), NADH2-P2 (B), NADH2-IP/NADH2-P2 (C), Src-IP (D) and NADH2-IP/SRC-IP (E). Filled circles, trained chicks. Open circle with error bars, mean level in untrained controls ± SEM; individual values for untrained chicks are shown as open circles along the y-axis. Vertical dashed lines, ‘no preference’ score 50 (approach to training and alternative stimuli equal/no learning) and ‘maximum preference’ score 100 (approach to training stimulus only/strong learning); horizontal dashed lines, y-intercepts for ‘no preference’ and ‘maximum preference’. Gray bars on *y*-axis, ± SE of intercept. For NADH2-IP, NADH2-IP/NADH2-P2 and NADH2-IP/Src-IP the correlations are significant. For NADH2-IP and NADH2-IP/NADH2-P2 the differences between the untrained mean and the ‘maximum preference’ intercept are also significant.

The correlation between amount of Src-bound NADH2 and preference score is significant (r = 0.72, P = 0.019, Fig 1A, Table 1). The ‘maximum preference’ intercept significantly exceeds the untrained mean (P = 0.010), which in turn does not differ significantly from the ‘no preference’ intercept. The variance about the regression line is not significantly lower than the variance of the untrained controls. These results indicate an association of NADH2-bound Src with preference score that is a consequence of learning during training (see Discussion).

**Table 1:** Standardised relative amount of protein. Summary of results for the left IMM 1 h after the end of training for: NADH2-IP, NADH2-P2, NADH2-IP /NADH2-P2, Src-IP and NADH2-IP/Src-IP. Data for untrained chicks in upper part of the table and data from trained chicks below. y-intercepts for ‘no preference’ and ‘maximum preference’ are given, with comparisons of intercepts with untrained values (*t*-tests). Bottom line: result of *F*-test comparing residual variance from the regression with variance of untrained controls. Statistically significant results are shown by asterisks.

Brain Region	Left IMM				
Protein	NADH2-IP	NADH2-P2	NADH2-IP/NADH2-P2	SRC-IP	NADH2-IP/SRC-IP
	Untrained chicks				
Mean	0.58	0.87	0.67	0.83	0.72
SEM	0.010	0.032	0.027	0.050	0.047
DF	8	8	8	8	8
	Trained chicks				
Correlation protein amount vs preference score	0.72	-0.31	0.80	-0.009	0.66
DF	8	8	8	8	8
P	0.019*	0.39	0.005*	0.98	0.036*
y-intercept at preference score 100	0.74	0.86	0.86	0.90	0.82
SE y-intercept	0.049	0.048	0.048	0.042	0.058
	Comparison. y- intercept at preference score 100 vs mean for untrained chicks				
t	3.24	-0.19	3.44	1.10	1.38
DF	8.70	13.89	12.61	15.52	15.39
P	0.010*	0.85	0.0045*	0.29	0.19
y- intercept at preference score 50	0.51	0.92	0.56	0.90	0.57
SE of Y-intercept	0.053	0.051	0.053	0.047	0.063
	Comparison. y-intercept at preference score 50 vs mean for untrained chicks				
t	-1.21	0.85	-1.91	1.09	-1.84
DF	8.57	12.14	11.23	14.67	13.08
P	0.26	0.41	0.082	0.29	0.088
Residual regression variance/variance untrained	7.92	0.62	1.23	0.37	0.63
P	0.995	0.26	0.61	0.089	0.26

#### NADH2-P2

The correlation between amount of NADH2 in the P2 fraction and preference score is not significant (Fig 1B and Table 1); i.e. no learning-related changes are evident.

#### NADH2-IP/NADH2-P2

There is a significant correlation between preference score and the Src-bound proportion of total NADH2 (r = 0.80, P = 0.005, Fig 1C, Table 1). The difference between untrained mean and ‘maximum preference’ intercept is also significant (P = 0.0045). The untrained mean is statistically homogeneous with the ‘no preference’ intercept. The variance about the regression line is not significantly lower than the variance of the untrained controls. The learning-dependent change in amount of NADH2-IP (Fig 1A) is thus reflected in the proportion of total NADH2 bound to Src.

#### Src-IP

The correlation between amount of antibody-bound Src and preference score is not significant (Fig 1D and Table 1), indicating that the learning-related increase of Src-bound NADH2 is not attributable to a change in amount of Src in the P2 fraction. It is noteworthy that the amount of Src in homogenates from the left IMM 1 h after the end of training was previously found to be unassociated with preference score [6].

#### NADH2-IP/Src-IP

This ratio and preference score are significantly correlated (r = 0.66, P = 0.036, Fig 1E, Table 1), as might be expected from the results for the components of this ratio. However, the ‘maximum preference’ intercept and the mean of untrained controls are statistically homogeneous. The untrained mean, although nearly 20% higher than the ‘no preference’ intercept, is not significantly different from it. The variance about the regression plot is not significantly lower than the variance of untrained controls. The data thus indicate that the NADH2-IP/Src-IP ratio in the left IMM is associated with learning but that attainment of the highest preference score is insufficient to change this ratio from its level in untrained controls.

### Right IMM and PPN

**Fig 2:**
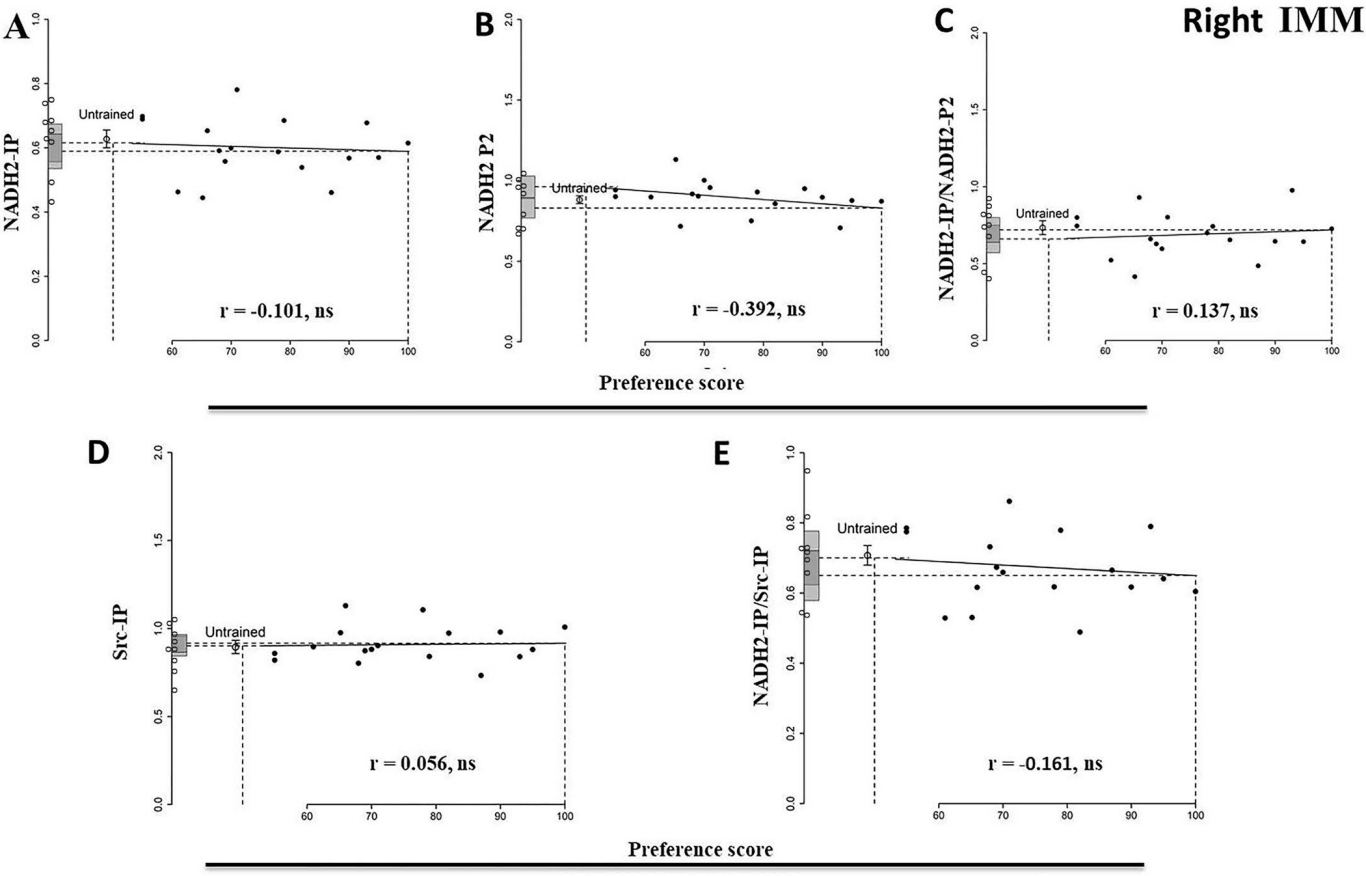
Right IMM 1 h after the end of training. Preference score plotted against standardized relative amount of NADH2-IP (A), NADH2-P2 (B), NADH2-IP/NADH2-P2 (C), Src-IP (D) and NADH2-IP/Src-IP (E). Plotting conventions and annotations as for Fig 1.

In these regions, no correlations are significant for any of the measures reported above and thus no learning-related changes are evident (Fig 2, S5, S6 Figs and S1–S3 Tables). In the right PPN the correlation between the amount of Src in immunoprecipitates and the preference score was borderline significant, (P = 0.05, S3 Table, S6 Fig), but the difference between the means of untrained controls and the ‘maximum preference’ intercept was not significant. There is thus no evidence that the amount of total NADH2 or of NADH2-IP in either side of the PPN covaries with learning.

### Conclusion 1

In the left IMM, the amount of NADH2 bound to Src was increased in a learning-related way independently of changes in total Src or total NADH2 in the P2 fraction. No such change was detected in the right IMM or on either side of the PPN.

**Twenty-four hours after the end of training**.

### Left IMM

#### NADH2-IP

**Fig 3:**
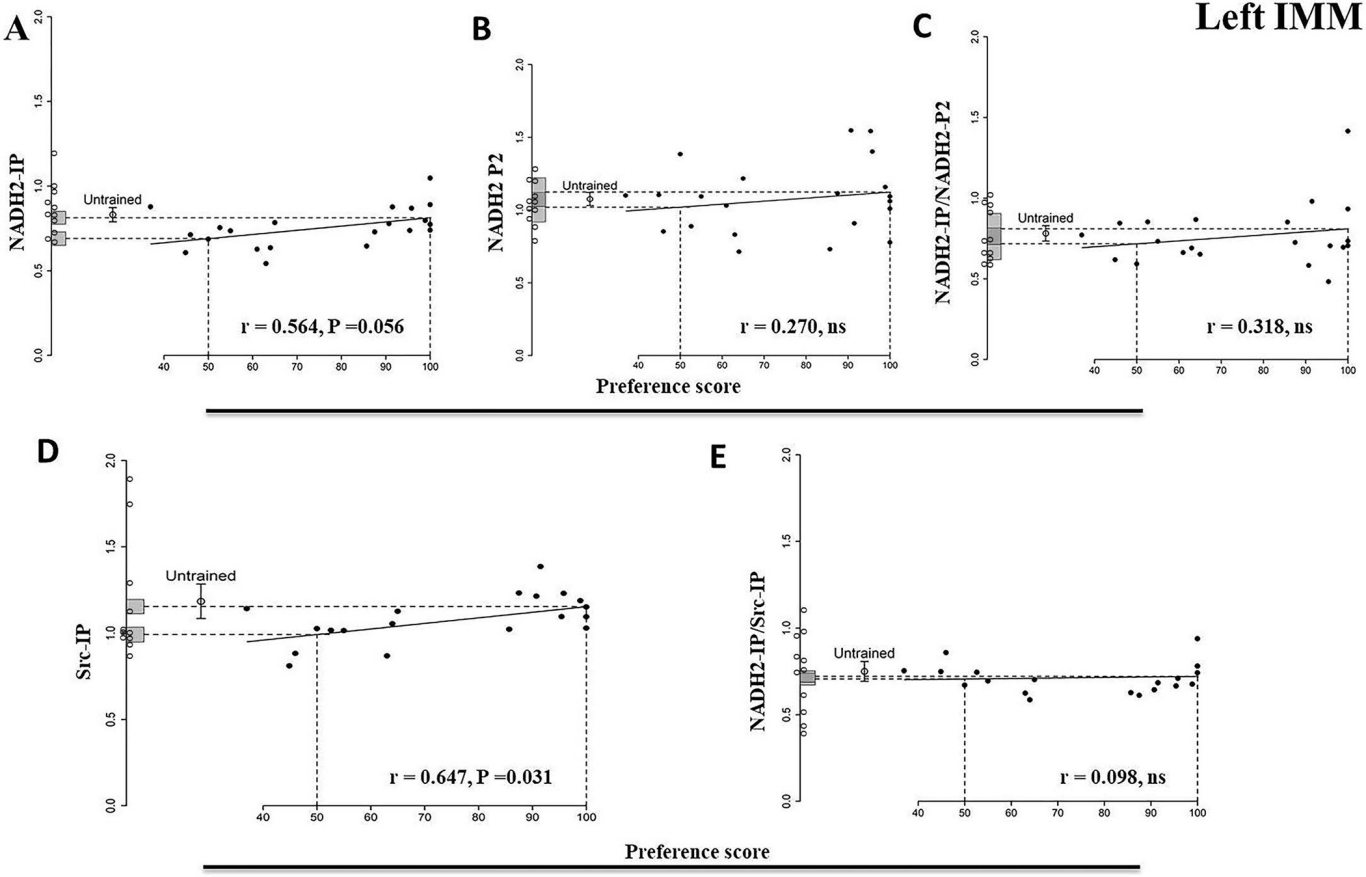
Left IMM 24 h after the end of training. Preference score plotted against standardized relative amounts of NADH2-IP (A), NADH2-P2 (B), NADH2-IP/NADH2-P2 (C), Src-IP (D) and NADH2-IP/Src-IP (E). Plotting conventions and annotations as for Fig 1. For Src-IP the correlation is significant.

The correlation with preference score was not significant (P = 0.055, Table 2, Fig 3A).

**Table 2:** Standardised relative amount of protein. Summary of results for the left IMM 24 h after the end of training for: NADH2-IP, NADH2-P2, NADH2-IP/NADH2-P2, Src-IP and NADH2-IP/SRC-IP. Conventions as for Table 1.

Brain Region	Left IMM				
Protein	NADH2-IP	NADH2-P2	NADH2-IP/NADH2-P2	SRC-IP	NADH2-IP/SRC-IP
Untrained chicks					
Mean	0.83	1.075	0.78	1.18	0.75
s.e.m	0.041	0.045	0.047	0.099	0.057
Df	10	10	10	10	10
Trained chicks					
Correlation protein amount vs preference score	0.56	0.27	0.32	0.65	0.098
Df	10	10	10	9	9
P	0.055	0.40	0.31	0.031*	0.77
y-intercept at preference score 100	0.81	1.12	0.81	1.15	0.72
SE y-intercept	0.037	0.098	0.090	0.041	0.033
Comparison. y- intercept at preference score 100 vs mean for untrained chicks					
T	-0.31	0.44	0.26	-0.28	-0.44
Df	19.81	14.01	14.58	13.32	15.90
P	0.76	0.67	0.80	0.78	0.67
y- intercept at preference score 50	0.69	1.020	0.717	0.991	0.705
SE of Y-intercept	0.039	0.10	0.10	0.042	0.034
Comparison. y- intercept at preference score 50 vs mean for untrained chicks					
T	-2.44	-0.50	-0.59	-1.78	-0.66
Df	18.21	13.19	13.65	18.65	18.22
P	0.025	0.63	0.57	0.092	0.52
Residual regression variance/variance untrained	0.69	2.31	1.15	0.14	0.28
P	0.28	0.90	0.59	0.003*	0.032

#### NADH2-P2

There was no significant correlation with preference score (Table 2, Fig 3B).

#### NADH2-IP/NADH2-P2

There was no significant correlation with preference score (Table 2, Fig 3C).

#### Src-IP

The amount of Src correlated significantly with preference score (P = 0.031, Table 2, Fig 3D). Neither the ‘maximum preference’ nor the ‘no preference’ intercept differed significantly from the untrained control mean value. This positive correlation for Src in immunoprecipitates is in agreement with the previous finding that the amount of total Src in homogenates from the IMM increased significantly with preference score 24 h after training [6].

#### NADH2-IP/Src-IP

This ratio was not significantly correlated with preference score (Table 2, Fig 3E), suggesting that the nearly significant correlation between preference score and NADH2 bound to Src (Fig 3A) is attributable to an increase in the amount of Src.

### Right IMM

#### NADH2-IP

**Fig 4:**
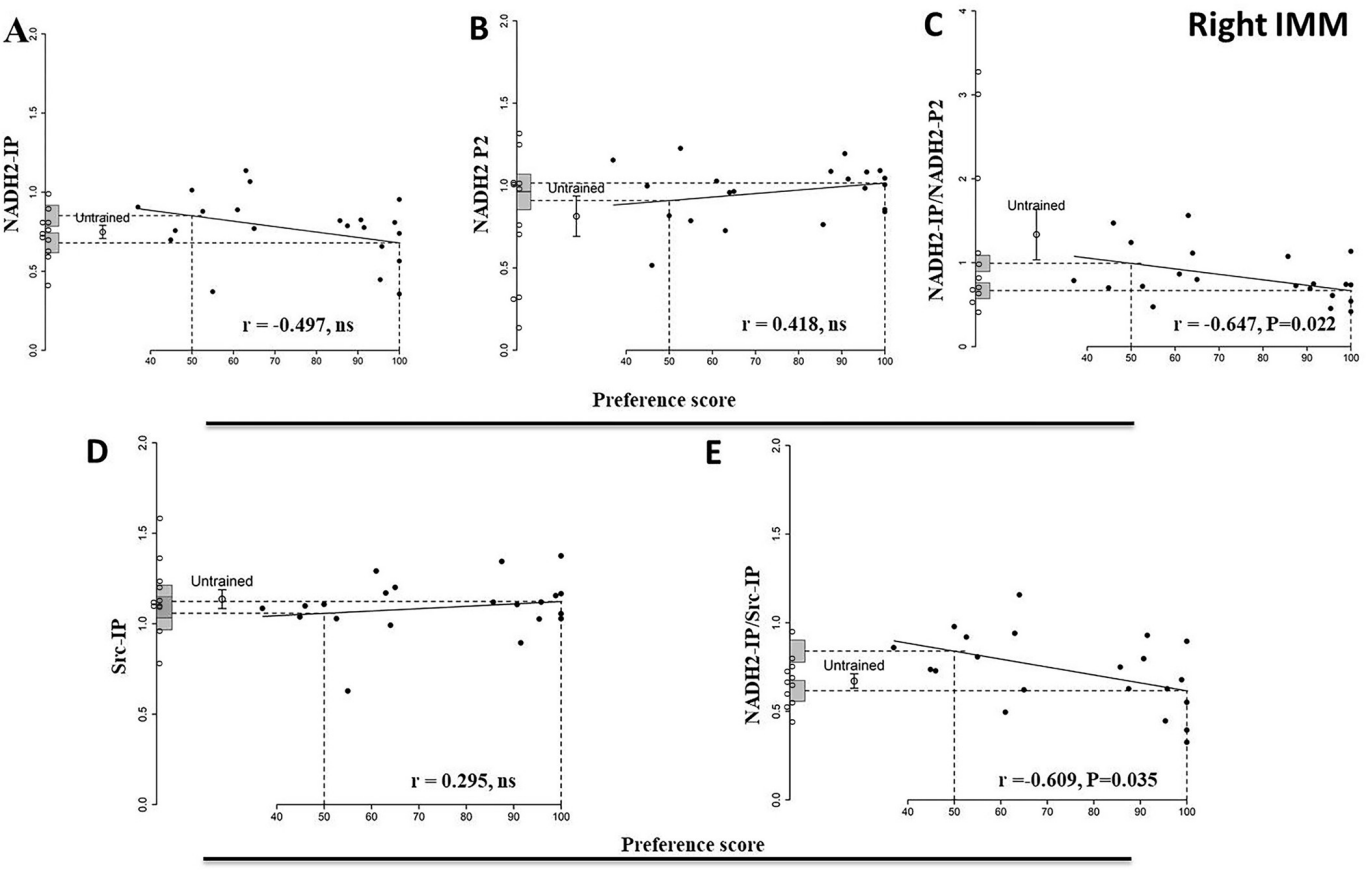
Right IMM 24 h after the end of training. Preference score plotted against standardized relative amounts of NADH2-IP (A), NADH2-P2 (B), NADH2-IP/NADH2-P2 (C), Src-IP (D) and NADH2-IP/Src-IP (E). Conventions and annotations as for Fig 1. For NADH2-IP/Src-IP the correlation is significant.

The correlation of Src-bound NADH2 with preference score is not significant (Fig 4A, Table 3).

**Table 3:** Standardised relative amount of protein. Summary of results for the Right IMM 24 h after the end of training for the following proteins and their ratios of NADH2-IP, NADH2-P2, NADH2-IP/NADH2-P2, Src-IP and NADH2-IP/SRC-IP. Conventions as for Table 1.

Brain Region	Right IMM				
Protein	NADH2-IP	NADH2-P2	NADH2-IP/NADH2-P2	Src-IP	NADH2-IP/Src-IP
Untrained chicks					
Mean	0.75	0.81	1.34	1.14	0.67
s.e.m	0.041	0.12	0.30	0.051	0.041
Df	10	10	10	10	10
Trained chicks					
Correlation protein amount vs preference score	-0.50	0.42	-0.65	0.30	-0.61
Df	10	10	10	10	10
P	0.099	0.18	0.022*	0.35	0.035*
y-intercept at preference score 100	0.68	1.015	0.67	1.12	0.62
SE y-intercept	0.063	0.054	0.093	0.090	0.059
Comparison. y- intercept at preference score 100 vs mean for untrained chicks					
T	-0.90	1.51	-2.11	-0.12	-0.76
Df	17.35	13.76	11.91	15.89	17.86
P	0.38	0.15	0.056	0.90	0.46
y- intercept at preference score 50	0.85	0.91	0.992	1.057	0.84
SE of Y-intercept	0.066	0.056	0.097	0.092	0.062
Comparison. y- intercept at preference score 50 vs mean for untrained chicks					
T	1.30	0.71	-1.08	-0.74	2.26
Df	15.24	19.82	19.95	14.55	15.54
P	0.21	0.49	0.29	0.47	0.038*
Residual regression variance/variance untrained	1.85	0.13	0.056	0.56	1.87
P	0.83	0.001*	4.59E-05*	0.18	0.83

#### NADH2-P2

There was no significant correlation between total amount of NADH2 in the P2 fraction and preference score (Fig 4B, Table 3).

#### NADH2-IP/NADH2-P2

There is a significant negative correlation with preference score in the right IMM (r = -0.64, P = 0.022, Fig 4C, Table 3). The mean of the untrained group, though higher than the ‘maximum preference’ intercept, is not significantly so (P = 0.056). The ‘no preference’ intercept does not differ significantly from the untrained mean value. The variance about the regression line is significantly lower than the variance of the untrained chicks, consistent with a predisposition to learn rather than with a consequence of learning.

#### Src-IP

The correlation with preference score is not significant (Fig 4D, Table 3).

#### NADH2-IP/Src-IP

This ratio is negatively correlated with preference score (r = -0.60, P = 0.035, Fig 4E, Table 3). The ‘no preference’ intercept is significantly higher than the mean of untrained chicks (P = 0.038, Fig 4E, Table 3) but the ‘maximum preference’ intercept does not differ significantly from the untrained mean value. The variance about the regression line does not differ significantly from the variance for untrained controls.

The plot of NADH2-IP/Src-IP resembles that of 416P-Src/Total Src in the right IMM 24 h after training observed in our previous study [6]. In both cases, the regression is significantly negative, but for NADH2-IP/Src-IP the correlation indicates a result of learning, whereas for 416P-Src/Total Src it suggests a predisposition to learn. 416P-Src is an activated form of Src. If the Src-NADH2 complex is also an active form of the enzyme, results from both studies point to decreased activity of some forms of Src in the right IMM 24 h after training, reflecting a predisposition to learn.

#### Left PPN and Right PPN

For none of the measurements studied was the correlation with preference score significant (S4 and S5 Tables, S7 and S8 Figs).

### Conclusion 2

In contrast to the results at 1 h, only in the right IMM was there a correlation with preference score for which an intercept is significantly different from the untrained value. This applies to the Src-bound NADH2/Src ratio, which is decreased in a learning-related way independently of changes in total Src or total NADH2 in the P2 fraction. Learning-related changes in Src-bound NADH2 thus depend on time after training and brain region.

## Discussion

We have elucidated the role of Src-NADH2 complexes in the memory of visual filial imprinting in chicks. Our results demonstrate learning-related changes in these complexes, which vary between brain regions and change as memory consolidates. Our experiments exclude artificial, random formation of complexes and, when taken together with previous results [6], indicate a multifaceted involvement of Src in learning and memory processes.

A learning-related change in NADH2-Src complex was observed 1 h after the end of training. At this time, the amount of Src-bound NADH2 bound was increased in a learning-related way. This increase is not the result of a change in total NADH2 or Src–it specifically reflects the increased amount of the Src-NADH2 complex, and is explicable as a result of learning rather than an increased capacity to learn. Notably, in both the present results and those of our previous experiments at the same time point [6] there were no changes in total Src. These two independent series of experiments demonstrate that, at early stages after training, there are no changes in the total amount of Src but that this multifunctional enzyme is implicated in learning by its activity changes (regulated by phosphorylation at Tyr-416 and Tyr-527; see [6]) or by complex formation with NADH2. As our study has shown, NADH2 interacts both with activated and inhibited forms of Src and changes in Src-NADH2 complexes could potentially involve both forms of the enzyme.

At 24 h after training there is a learning-related increase with preference score in the total amount of Src [6] in both left and right IMM. In the present experiments, the correlation for total Src from the P2 fraction in the left IMM is also positive and significant, but not in the right IMM, where the correlation was positive but not significant. The dominant change at 24 h is therefore an increase in the total amount of Src, which is a major contributor to the correlations with preference score shown by the ratios of which it is a component. In the left IMM, the correlation for Src-bound NADH2 is positive and significant using a one-tailed test (P = 0.028) but the correlation of NADH2-Src with preference score very low (r = 0.01, see Table 2), due to parallel increase in the total amount of Src. In the right IMM the trend of the correlation for Src-bound NADH2 is negative and with the parallel increase in the total amount of Src it yields a significant negative correlation of NADH2/Src ratio. In our previous study [6] in the right IMM 24 h after training, a negative correlation was also observed for 416P-Src/Total Src, which is also mainly due to the increase in the amount of total Src. In sum, data obtained in our previous and present study indicate that at early times after imprinting training there are learning-related functional changes of Src protein without changes in its total amount, whereas stable long-term recognition memory involves an increase in the total amount of Src, including its various modifications.

NADH2 is a mitochondrially encoded protein, a component of complex I in mitochondria, and interacts with Src outside this intracellular organelle at brain excitatory synapses. NADH2 is crucial for regulation of synaptic NMDA receptor activity by Src kinase [4]. NMDA receptors are crucially involved in visual imprinting in chicks, since imprinting is blocked by localized injection of an NMDA receptor antagonist into the left IMM [34]. From 6 h after the end of training, there is a learning-related increase in NMDA receptor ligand binding in the left IMM [22, 35]. Study of Ca^2+^-dependent, K^+^-evoked release of glutamate suggests that soon after chicks have been exposed to an imprinting stimulus, glutamatergic excitatory transmission in IMM is enhanced, and remains so for at least 24 h [36]. It seems likely that NADH2-Src complexes are also involved in this learning process.

With the exception of one correlation (NADH2-IP/NADH2-P2) in the right IMM 24 h after training, all significant correlations are attributable to a result of learning rather than to a predisposition to learn. By”predisposition to learn” we mean a capacity, occurring in the absence of training, to readily learn the characteristics of the training stimulus. This capacity will differ between individuals by natural variation: for whatever reason, some chicks are capable of learning better than others. This variation can be responsible for a correlation with preference score, and may be detected when training occurs and the residual variance about the regression line is significantly lower than the variance in the untrained controls. Such a predisposition presumably imparts a survival benefit: when a chick is exposed to an imprinting stimulus, it will become more strongly imprinted to the extent that it possesses this predisposition. Chicks express other predispositions of course, such as their extensively studied preference for animacy [37].

The functional significance of the hemispheric asymmetry observed in the present experiments is not yet known. However, the asymmetry is paralleled not only by other biochemical asymmetries, but also by certain electrophysiological asymmetries; it will be instructive in future to investigate possible associations between these separate findings. The proportion of microelectrode recording sites responsive to the alternative stimulus is significantly less in the right IMM than in the left IMM and the ratio of excitatory to inhibitory responses to the red box and the blue cylinder is significantly higher in the right than in the left IMM [38]. The ability of neurons in the right IMM to discriminate between familiar and unfamiliar chicks was found to be significantly greater than in the left IMM [39], consistent with suggestions that the right hemisphere is specialised in detecting novelty [40, 41], including discrimination between familiar and unfamiliar individuals [42–47]. It has also been suggested that the right hemisphere has a dominant role in the early stages of imprinting recall [48].

Functional differences between the two sides of the IMM reflect molecular processes involved in the learning and memory of visual imprinting. Right and left sides are not different for some changes at the early time after training (Fos immunoreactivity, CaMKII, see [23, 29], whereas at 24 h after training learning-related biochemical changes are usually more strongly expressed in the left IMM than in the right IMM [6, 11, 14, 20, 21]. This is not the case for the present data; at 1 h after the end of training, the hemispheric asymmetry is clearly expressed by learning-related up-regulation of NADH2-Src complexes in the left IMM, whereas the learning related changes at 24h after imprinting takes place only in the right IMM.

Learning-related changes in NADH2-Src complexes were found in the left or right IMM and not in the PPN, a control forebrain region not involved in the memory of visual imprinting. These results once more reflect a regional specificity found in all our previous studies of imprinting in which measurements have been made on these two regions; see e.g. [14].

## Materials and methods

### Behavioral training and testing

Details of chick strains used in experiments, hatching, behavioral training and testing, and tissue sample removal are given in Supplementary Materials (S1 File “Chick training and apparatus”) and in previous publications [6, 21].

Twenty batches of eggs were used, each batch comprising fertilized eggs incubated simultaneously. Behavioral experiments were conducted at the I. Beritashvili Centre of Experimental Biomedicine, in compliance with its approved animal care policy. Experimental design was approved by the Bioethics Committee of the I. Beritashvili Centre of Experimental Biomedicine (Protocol N03/09.12.2021). The sample sizes used have previously been found sufficient for reliable detection of molecular changes attributable to learning.

Tissue was collected immediately after decapitation, either 1 h or 24 h after the end of training. Each chick contributed four pieces of tissue, from the left and right IMM and the left and right PPN, which were frozen immediately on dry ice. Each replicate of an experiment was performed within a batch, each batch yielding tissue from up to four chicks (one untrained and up to three trained). There were thus up to 16 samples per batch. Samples were taken from 18 trained chicks and 9 untrained controls 1 h after the end of training, and from 22 trained chicks and 11 untrained controls 24 h after training.

The locations of the IMM and PPN are published [17]. Details of the IMM removal procedure are provided in Davies et al. [49] and details of PPN removal in Solomonia et al. [26]. Samples were coded after collection and all further procedures performed blind.

### Antibody preparation

Rabbit polyclonal antibodies against chicken mitochondrial NADH2 (Uniprot P18937) were generated by Abbiotec to our special order. The antibodies were generated against the 20-amino acid sequence TITLPPNSSNHMKLWRTNKTC (positions 301–320 of NADH2 plus a cysteine residue for attachment of keyhole limpet hemocyanin (KLH)). Custom rabbit polyclonal antibodies against chicken Src (Uniprot P00523) were produced in a similar way (see also [6]). The peptide RRSLEPPDSTHHGGFPASC was used as antigen (amino acid residues 15–32 of chicken Src plus a terminal cysteine for conjugation to KLH). This epitope does not correspond to known phosphorylation sites. The antibody is therefore assumed to recognize both phosphorylated and non-phosphorylated forms. Antibodies were purified on antigen affinity columns and specificity confirmed with adsorption by the target peptide.

### Preparation of subcellular fraction for immunoaffinity (IA) chromatography

Tissue samples were rapidly homogenized in 20 mM Tris-HCl (pH 7.4), 0.32 M sucrose, 1 mM ethylenediaminetetraacetic acid, 1 mM sodium orthovanadate, 10 mM sodium pyrophosphate, 0.5 mM methyleneglycol-bis(2-aminoethylether)-N,N,N′,N′-tetraaceticacid and a cocktail of protease inhibitors (Sigma, P8340), and centrifuged at 1000 g for 10 min. One third of the supernatant was centrifuged at 15,000 g for 20 min. The resulting pellet was washed once and dissolved in 5% sodium dodecylsulphate (SDS) and is referred to as the P2 mitochondrial-membrane fraction. The rest of the supernatant was centrifuged also at 15,000 g for 20 min and the resulting pellet solubilized in RIPA buffer (50 mM Tris-HCl pH 7.6, 150 mM NaCl, 1 mM ethylenediaminetetraacetic acid, 1% Nonidet P-40, 2.5 mg/ml sodium deoxycholate, 1 mM Na_3_VO_4_) and a cocktail of protease inhibitors (Sigma, P8340) for two hours at 4°C with periodic stirring.

### Immunoafinity (IA) chromatography

Attachment of Src antibody or antibody against NADH2 protein (10 μg of each antibody) to a protein A/G column was carried out with a Pierce Crosslink Immunoprecipitation kit (Product No. 26147) according to the manufacturer’s instructions. During this procedure, antibody is irreversibly attached to agarose beads so that co-elution of antibody with the purified protein is practically excluded [https://www.thermofisher.com/order/catalog/product/26147].

The RIPA buffer-solubilized membrane mitochondrial fraction was pre-cleared on Control Agarose resin and protein concentrations were determined in quadruplicate using a micro bicinchoninic acid protein assay kit (Pierce). Samples containing 100 μg protein from the same batch were loaded onto the columns of protein A/G agarose cross-linked to the polyclonal antibody against Src (10 μg antibody). The columns were incubated overnight at 4°C with periodic stirring. Proteins non-specifically bound to the A/G agarose were removed by washing with RIPA buffer and then column-incubated with non-reducing sodium dodecyl sulfate (SDS) sample buffer at 60°C for 15 min. Columns were centrifuged and a concentrated solution of dithiothreitol was added up to a final concentration of 50 mM to eluates and heated at 95°C for 5 min. The whole eluates (35 μl) were loaded on each line for electrophoresis.

The IA chromatography on protein A/G agarose cross-linked with polyclonal antibodies against NADH2 were carried out exactly in the same way as described above.

To determine if our experimental conditions of IA chromatography accurately measured quantitative changes in Src-NADH2 complexes, we loaded four different amounts (50, 100, 150 and 200 μg of protein) of RIPA-soluble and Control Agarose Resin-precleared fractions on Src antibody protein A/G agarose columns. These amounts were chosen according to the protein amount (100 μg) loaded on our Src antibody-conjugated column. The chromatography and elution was carried out as described above.

### Electrophoresis and Western immunoblotting

Samples of P2 membrane fractions (30 μg) and proteins bound specifically to Src antibody columns (whole eluate) were subjected to SDS gel electrophoresis and western blotting as described in our previous publication [18]. After protein transfer to nitrocellulose membrane by western blotting, each membrane was cut into two parts for separate treatments by antibodies against Src and NADH2.

The affinity eluates from NADH2 antibodies linked to protein A/G agarose were immunostained with antibodies against Src, 416P-Src (Abcam, ab4816), and 527P-Src (Abcam, ab4817).

Standard immunochemical procedures were applied for the visualization of the target protein bands as described in e.g. [6]. Autoradiographs were calibrated using standard amounts of protein from P2 fractions of the IMMs of untrained chicks. The optical densities for these standards accurately reflected protein amount (S1 Fig). Optical density of experimental bands was expressed as a fraction of that for 30 μg of the protein standard [18] to give what will be referred to as “relative amount of protein”. To remove variation due to differences between batches, the batch mean was subtracted from the relative amount of protein for each sample and this difference then added to the overall mean of all batches. This quantity is referred to as “standardized relative amount of protein”.

Data from experimental stained protein bands were not normalized according to a housekeeping protein because one cannot *a priori* identify any such protein that is unaffected by imprinting [see for discussion 6, 18]. Instead, loading was controlled by Ponceau S staining and expression relative to protein standards as described above.

Images of all uncropped and unadjusted immunoblots are provided in S1 Raw images9.

### Statistical analysis

Statistical analysis was conducted as previously [6] by fitting a linear mixed-effects regression model to relative amount of protein, with fixed term preference score and random terms chick nested within batch. Data from the left and right sides of IMM and PPN were analysed separately because of their known functional disparity [14]. The R script used for statistical analysis is given in Supplementary Information of Margvelani et al.2018a [11] at https://doi.org/10.1038/s41598-018-35097-w.

### Criteria for learning-relatedness and predispositions

The following criteria were used to infer that a biochemical measure is learning-related: (i) a significant correlation with preference score; (ii) a level at maximum preference score (strong learning) significantly different from the mean for untrained chicks; (iii) no significant difference between the mean for untrained chicks and the level in chicks showing no learning despite being exposed to the training stimulus (estimated by the level at preference score 50). Such similarity between untrained chicks and non-learning chicks exposed to the training stimulus provides an important experimental control for side-effects of training such as locomotor activity, stress, handling, and other differences between the trained and untrained conditions that are confounded with learning.

Further analysis can indicate whether a result satisfying the above criteria is the result of learning, or whether it reflects a capacity to learn that was present before training. We refer to this second possibility as a predisposition to learn. In the event of a pure predisposition of this sort (i.e. no effect of learning during training) the total variance of the trained chicks is similar to the variance of the untrained controls since they are members of the same population. Additionally, the variance of the trained chicks is separable into a component due to the correlation and a second component (the residual variance about the regression line) that is necessarily lower than the variance of untrained chicks because the correlation component has been removed. Therefore, a residual variance about the regression line that is significantly lower than the variance in untrained chicks is evidence for a predisposition (see also Margvelani et al 2018 [11]). In contrast, where there is no significant reduction in residual variance, a significant change in a measurement at maximum preference score indicates that the correlation is a result of learning.

## Supporting information

S1 Table Standardised relative amount of protein. Summary of results for the Right IMM 1 h after the end of training for the following proteins and their ratios of NADH2-IP, NADH2-P2, NADH2-IP/NADH2-P2, Src-IP and NADH2-IP/SRC-IP.
(PDF)

S2 Table Standardised relative amount of protein. Summary of results for the Left PPN 1 h after the end of training for the following proteins and their ratios of NADH2-IP, NADH2-P2, NADH2-IP/NADH2-P2, Src-IP and NADH2-IP/SRC-IP.
(PDF)

S3 Table Standardised relative amount of protein. Summary of results for the Right PPN 1 h after the end of training for the following proteins and their ratios of NADH2-IP, NADH2-P2, NADH2-IP/NADH2-P2, Src-IP and NADH2-IP/SRC-IP.
(PDF)

S4 Table Standardised relative amount of protein. Summary of results for the Left PPN 24 h after the end of training for the following proteins and their ratios of NADH2-IP, NADH2-P2, NADH2-IP/NADH2-P2, Src-IP and NADH2-IP/SRC-IP.
(PDF)

S5 Table Standardised relative amount of protein. Summary of results for the Right PPN 24 h after the end of training for the following proteins and their ratios of NADH2-IP, NADH2-P2, NADH2-IP/NADH2-P2, Src-IP and NADH2-IP/SRC-IP.
(PDF)

S1 Fig Sample films and calibration plots for: (A) NADH2-IP; (B) Src-IP and (C) NADH2-P2. On all sample films: 1-Left IMM, Good learner chick; 2-Right IMM Good learner chick; 3-Left PPN Good learner chick; 4- Right PPN Good Learner chick; 5-Left IMM, Poor learner chick; 6-Right IMM Poor learner chick; 7-Left PPN Poor learner chick; 8- Right PPN Poor Learner chick; 9-Left IMM, Untrained chick; 10-Right IMM Untrained chick; 11-Left PPN Untrained chick; 12- Right PPN Untrained chick. All sample gels contains internal standards–IS1, IS2, IS3,IS4 containing, respectively, 15, 30, 45, and 60 μg protein and corresponding to 0.5, 1.0, 1.5 and 2.0 relative amounts of protein respectively. For each calibration the r^2^ value was more than 97%. Note that the effects reported in this paper were obtained from the complete dataset and are not necessarily apparent from visual inspection of a single gel.
(TIF)

S2 Fig The specificity of NADH2 and Src staining—confirmation with blocking peptides. A-Western immunoblot image for Src staining. Lanes 1,2 –immunostaining with Src antibodies, Lanes 3,4 –immunostaining with Src antibodies containing corresponding antigen peptide. B- A-Western immunoblot image for NADH2 staining. Lanes 5,6 –immunostaining with NADH2 antibodies, Lanes 7,8 –immunostaining with NADH2 antibodies containing corresponding antigen peptide.
(TIF)

S3 Fig Western immunoblot images and regression plots of Src and NADH2 immunoreactivity of IA chromatography affinity eluate fractions. Four different amounts (50, 100, 150 and 200 μg of protein) of RIPA buffer-solubilized and Protein A/G agarose-precleared fractions were applied to Src antibodies immobilized on columns. The Western blots of eluates were stained with antibodies against Src and NADH2 proteins. A-NADH2 immunostaining; B-regression plot of NADH2 immunoreactivity with the amount of loaded protein on IA chromatography; C-Src immunostaining; D-regression plot of Src immunoreactivity with the amount of loaded protein on IA chromatography. For both Src and NADH2, least-squares regression showed a virtually perfect fit to a straight line.
(TIF)

S4 Fig Western immunoblot images of IA chromatography on NADH2 antibodies column. The blots were stained with antibodies against: 416P-Src (A), 527P-Src (B) and total Src (C). Both phosphorylated forms of Src are interacting with NADH2.
(TIF)

S5 Fig Left PPN 1 h after the end of training. Preference score plotted against standardized relative amounts of NADH2-IP (A), NADH2-P2 (B), NADH2-IP/NADH2-P2 (C), Src-IP (D) and NADH2-IP/SRC-IP (E).Conventions otherwise as for Fig 1. No correlation was significant.
(TIF)

S6 Fig Right PPN 1 h after the end of training. Preference score plotted against standardized relative amounts of NADH2-IP (A), NADH2-P2 (B), NADH2-IP/NADH2-P2 (C), Src-IP (D) and NADH2-IP/SRC-IP (E). Conventions otherwise as for Fig 1. No correlation was significant.
(TIF)

S7 Fig Left PPN 24 h after the end of training. Preference score plotted against standardized relative amounts of NADH2-IP (A), NADH2-P2 (B), NADH2-IP/NADH2-P2 (C), Src-IP (D) and NADH2-IP/SRC-IP (E).Conventions otherwise as for Fig 1. No correlation was significant.
(TIF)

S8 Fig Right PPN 24 h after the end of training. Preference score plotted against standardized relative amounts of NADH2-IP (A), NADH2-P2 (B), NADH2-IP/NADH2-P2 (C), Src-IP (D) and NADH2-IP/SRC-IP (E). Conventions otherwise as for Fig 1. No correlation was significant.
(TIF)

S1 Raw images Images for all uncropped and unadjusted blots. (PDF)

S1 File “Chick training and apparatus”. (DOCX)
