# Supplementary material for: Src-NADH dehydrogenase subunit 2 complex and recognition memory of imprinting in domestic chicks
Source: PLoS One. 2024 Jan 29;19(1):e0297166. doi: 10.1371/journal.pone.0297166 (PMC10824410; doi:10.1371/journal.pone.0297166)
Supplement: S1 Table — Summary of results for the Right IMM 1 h after the end of training for the following proteins and their ratios of NADH2-IP, NADH2-P2, NADH2-IP/NADH2-P2, Src-IP and NADH2-IP/SRC-IP. (PDF) [file pone.0297166.s001.pdf]

S1 Table. Standardised relative amount of protein. Summary of results for the Right IMM 1h after the end of training for the following proteins and their ratios of NADH2-IP, NADH2-P2, NADH2-IP /NADH2-P2, Src-IP and NADH2-IP/SRC-IP

Brain Region	Right IMM				
Protein	**NADH2-IP**	**NADH2-P2**	**NADH2-IP/NADH2-P2**	**SRC-IP**	**NADH2-IP/SRC-IP**
Untrained chicks					
Mean	0.63	0.88	0.73	0.89	0.71
s.e.m	0.03	0.02	0.04	0.04	0.03
Df	8	8	8	8	8
Trained chicks					
Correlation protein amount vs preference score	-0.10	-0.39	0.14	0.06	-0.16
Df	8	8	8	8	8
P	0.78	0.26	0.71	0.88	0.66
y-intercept at preference score 100	0.59	0.83	0.72	0.92	0.65
SE y-intercept	0.05	0.06	0.08	0.050	0.07
Comparison. y- intercept at preference score 100 vs mean for untrained chicks					
T	-0.63	-0.79	-0.16	0.34	-0.75
Df	11.96	10.60	12.47	14.75	10.42
P	0.54	0.45	0.88	0.74	0.47
y- intercept at preference score 50	0.62	0.96	0.66	0.90	0.70
SE of Y-	0.06	0.07	0.09	0.06	0.08

intercept					
Comparison. y- intercept at preference score 50 vs mean for untrained chicks					
T	-0.19	1.13	-0.73	0.10	-0.09
Df	10.83	9.90	11.07	12.51	9.87
P	0.85	0.29	0.48	0.93	0.93
Residual regression variance/variance untrained	1.48	2.94	1.72	0.96	2.03
P	0.70	0.93	0.77	0.48	0.83

Data for untrained chicks are in the upper part of the table and data from trained chicks below. y-intercepts for preference scores 50 and 100 are given, together with results of comparisons of these intercepts with mean values for untrained chicks using t-tests. On the bottom line is given the probability (F-test) for a comparison of residual variance from the regression with the variance of untrained chicks. Asterisks indicate statistically significant results.
