# Supplementary material for: Src-NADH dehydrogenase subunit 2 complex and recognition memory of imprinting in domestic chicks
Source: PLoS One. 2024 Jan 29;19(1):e0297166. doi: 10.1371/journal.pone.0297166 (PMC10824410; doi:10.1371/journal.pone.0297166)
Supplement: S2 Table — Summary of results for the Left PPN 1 h after the end of training for the following proteins and their ratios of NADH2-IP, NADH2-P2, NADH2-IP/NADH2-P2, Src-IP and NADH2-IP/SRC-IP. (PDF) [file pone.0297166.s002.pdf]

S2 Table. Standardised relative amount of protein. Summary of results for the left PPN 1h after the end of training for the following proteins and their ratios of NADH2-IP, NADH2-P2, NADH2-IP /NADH2-P2, Src-IP and NADH2-IP/SRC-IP

Brain Region	Left PN				
Protein	**NADH2-IP**	**NADH2-P2**	**NADH2-IP/NADH2-P2**	**SRC-IP**	**NADH2-IP/SRC-IP**
Untrained chicks					
Mean	0.56	0.76	0.88	0.86	0.65
s.e.m	0.04	0.07	0.21	0.04	0.04
Df	8	8	8	8	8
Trained chicks					
Correlation protein amount vs preference score	0.46	-0.48	0.60	0.50	-0.18
Df	8	7	7	8	8
P	0.18	0.19	0.09	0.14	0.62
y-intercept at preference score 100	0.68	0.59	1.23	1.03	0.65
SE y-intercept	0.08	0.07	0.17	0.10	0.23
Comparison. y- intercept at preference score 100 vs mean for untrained chicks					
T	1.44	-1.72	1.27	1.58	0.01
Df	11.87	15	14.66	10.41	8.60
P	0.18	0.11	0.22	0.14	0.99
y- intercept at preference score 50	0.52	0.73	0.59	0.74	0.87
SE of Y-intercept	0.08	0.07	0.20	0.11	0.25

Comparison. y- intercept at preference score 50 vs mean for untrained chicks					
T	-0.39	-0.36	-1.01	-1.10	0.84
Df	10.87	12.87	13.22	9.76	8.47
P	0.70	0.73	0.33	0.30	0.42
Residual regression variance/variance untrained	1.01	0.21	0.34	3.41	13.55
P	0.50	0.03	0.08	0.95	0.10

Data for untrained chicks are in the upper part of the table and data from trained chicks below. y-intercepts for preference scores 50 and 100 are given, together with results of comparisons of these intercepts with mean values for untrained chicks using t-tests. On the bottom line is given the probability (F-test) for a comparison of residual variance from the regression with the variance of untrained chicks. Asterisks indicate statistically significant results.
