# Supplementary material for: Src-NADH dehydrogenase subunit 2 complex and recognition memory of imprinting in domestic chicks
Source: PLoS One. 2024 Jan 29;19(1):e0297166. doi: 10.1371/journal.pone.0297166 (PMC10824410; doi:10.1371/journal.pone.0297166)
Supplement: S3 Table — Summary of results for the Right PPN 1 h after the end of training for the following proteins and their ratios of NADH2-IP, NADH2-P2, NADH2-IP/NADH2-P2, Src-IP and NADH2-IP/SRC-IP. (PDF) [file pone.0297166.s003.pdf]

S3 Table. Standardised relative amount of protein. Summary of results for the Right PPN 1h after the end of training for the following proteins and their ratios of NADH2-IP, NADH2-P2, NADH2-IP /NADH2-P2, Src-IP and NADH2-IP/SRC-IP

Brain Region	Right PN				
Protein	**NADH2-IP**	**NADH2-P2**	**NADH2-IP/NADH2-P2**	**SRC-IP**	**NADH2-IP/SRC-IP**
Untrained chicks					
Mean	0.55	0.78	0.70	0.87	0.63
s.e.m	0.04	0.05	0.07	0.03	0.05
Df	7	8	7	8	7
Trained chicks					
Correlation protein amount vs preference score	0.33	0.02	0.19	0.63	0.14
Df	8	8	8	8	8
P	0.35	0.96	0.60	0.05*	0.70
y-intercept at preference score 100	0.58	0.75	0.78	0.88	0.69
SE y-intercept	0.04	0.06	0.09	0.04	0.06
Comparison. y- intercept at preference score 100 vs mean for untrained chicks					
T	0.41	-0.46	0.78	0.27	0.74
Df	14.95	15.56	14.45	15.20	14.66
P	0.69	0.65	0.45	0.79	0.47
y- intercept at preference score 50	0.50	0.74	0.70	0.75	0.64
SE of Y-intercept	0.05	0.06	0.10	0.04	0.07

Comparison. y- intercept at preference score 50 vs mean for untrained chicks					
T	-0.90	-0.50	-0.01	-2.22	0.16
Df	13.14	13.18	12.51	13.05	12.72
P	0.38	0.63	0.99	0.044*	0.88
Residual regression variance/variance untrained	0.76	0.74	1.01	0.43	0.92
P	0.35	0.34	0.50	0.13	0.45

Data for untrained chicks are in the upper part of the table and data from trained chicks below. y-intercepts for preference scores 50 and 100 are given, together with results of comparisons of these intercepts with mean values for untrained chicks using t-tests. On the bottom line is given the probability (F-test) for a comparison of residual variance from the regression with the variance of untrained chicks. Asterisks indicate statistically significant results.
