# Supplementary material for: Src-NADH dehydrogenase subunit 2 complex and recognition memory of imprinting in domestic chicks
Source: PLoS One. 2024 Jan 29;19(1):e0297166. doi: 10.1371/journal.pone.0297166 (PMC10824410; doi:10.1371/journal.pone.0297166)
Supplement: S4 Table — Summary of results for the Left PPN 24 h after the end of training for the following proteins and their ratios of NADH2-IP, NADH2-P2, NADH2-IP/NADH2-P2, Src-IP and NADH2-IP/SRC-IP. (PDF) [file pone.0297166.s004.pdf]

S4 Table. Standardised relative amount of protein. Summary of results for the Left PPN 24h after the end of training for the following proteins and their ratios of NADH2-IP, NADH2-P2, NADH2-IP /NADH2-P2, Src-IP and NADH2-IP/SRC-IP

Brain Region	Left PN				
Protein	**NADH2-IP**	**NADH2-P2**	**NADH2-IP/NADH2-P2**	**SRC-IP**	**NADH2-IP/SRC-IP**
Untrained chicks					
Mean	0.65	0.87	0.77	1.07	0.67
s.e.m	0.04	0.07	0.06	0.07	0.10
Df	10	9	9	10	10
Trained chicks					
Correlation protein amount vs preference score	0.43	0.19	0.33	-0.28	0.41
Df	9	10	9	10	9
P	0.18	0.56	0.33	0.37	0.21
y-intercept at preference score 100	1.02	1.10	0.98	1.21	0.87
SE y-intercept	0.11	0.09	0.11	0.09	0.11
Comparison. y- intercept at preference score 100 vs mean for untrained chicks					
T	3.26	2.09	1.67	1.26	1.30
Df	11.01	18.78	13.19	19.51	18.22
P	0.01*	0.05*	0.12	0.22	0.21
y- intercept at preference score 50	0.79	1.02	0.81	1.30	0.63
SE of Y-intercept	0.11	0.09	0.11	0.09	0.11
Comparison. y- intercept at preference score 50 vs mean for untrained chicks					
T	1.17	1.33	0.25	2.01	-0.28
Df	10.79	16.56	12.40	16.98	15.65

P	0.27	0.20	0.80	0.06	0.79
Residual regression variance/variance untrained	7.29	1.37	3.64	0.58	1.12
P	0.99	0.68	0.97	0.20	0.57

Data for untrained chicks are in the upper part of the table and data from trained chicks below. y-intercepts for preference scores 50 and 100 are given, together with results of comparisons of these intercepts with mean values for untrained chicks using t-tests. On the bottom line is given the probability (F-test) for a comparison of residual variance from the regression with the variance of untrained chicks. Asterisks indicate statistically significant results.
