# Supplementary material for: Src-NADH dehydrogenase subunit 2 complex and recognition memory of imprinting in domestic chicks
Source: PLoS One. 2024 Jan 29;19(1):e0297166. doi: 10.1371/journal.pone.0297166 (PMC10824410; doi:10.1371/journal.pone.0297166)
Supplement: S5 Table — Summary of results for the Right PPN 24 h after the end of training for the following proteins and their ratios of NADH2-IP, NADH2-P2, NADH2-IP/NADH2-P2, Src-IP and NADH2-IP/SRC-IP. (PDF) [file pone.0297166.s005.pdf]

S5 Table. Standardised relative amount of protein. Summary of results for the Right PPN 24h after the end of training for the following proteins and their ratios of NADH2-IP, NADH2-P2, NADH2-IP /NADH2-P2, Src-IP and NADH2-IP/SRC-IP

Brain Region	Right PN				
Protein	**NADH2-IP**	**NADH2-P2**	**NADH2-IP/NADH2-P2**	**SRC-IP**	**NADH2-IP/SRC-IP**
Untrained chicks					
Mean	0.82	1.03	1.17	1.03	0.84
s.e.m	0.06	0.15	0.34	0.06	0.09
Df	10	9	9	9	9
Trained chicks					
Correlation protein amount vs preference score	0.59	0.44	-0.12	0.04	0.51
Df	9	9	8	8	7
P	0.06	0.17	0.75	0.92	0.16
y-intercept at preference score 100	0.83	1.23	0.72	1.11	0.88
SE y-intercept	0.052	0.11	0.082	0.11	0.083
Comparison. y- intercept at preference score 100 vs mean for untrained chicks					
T	0.056	1.08	-1.26	0.56	0.36
Df	18.70	16.82	10.04	12.50	15.96
P	0.96	0.29	0.24	0.59	0.72
y- intercept at preference score 50	0.73	1.03	0.75	1.09	0.74
SE of Y-intercept	0.05	0.11	0.08	0.11	0.08
Comparison. y- intercept at preference score 50 vs mean for untrained chicks					

T	-1.17	0.01	-1.18	0.48	-0.87
Df	17.33	17.13	16.88	11.79	13.88
P	0.26	0.99	0.25	0.64	0.40
Residual regression variance/variance untrained	0.17	0.30	0.02	1.36	0.36
P	0.006*	0.045*	6.29E-06*	0.673	0.093

Data for untrained chicks are in the upper part of the table and data from trained chicks below. y-intercepts for preference scores 50 and 100 are given, together with results of comparisons of these intercepts with mean values for untrained chicks using t-tests. On the bottom line is given the probability (F-test) for a comparison of residual variance from the regression with the variance of untrained chicks. Asterisks indicate statistically significant results.
