# Supplementary figures and images for: Src-NADH dehydrogenase subunit 2 complex and recognition memory of imprinting in domestic chicks
Source: PLoS One. 2024 Jan 29;19(1):e0297166. doi: 10.1371/journal.pone.0297166 (PMC10824410; doi:10.1371/journal.pone.0297166)

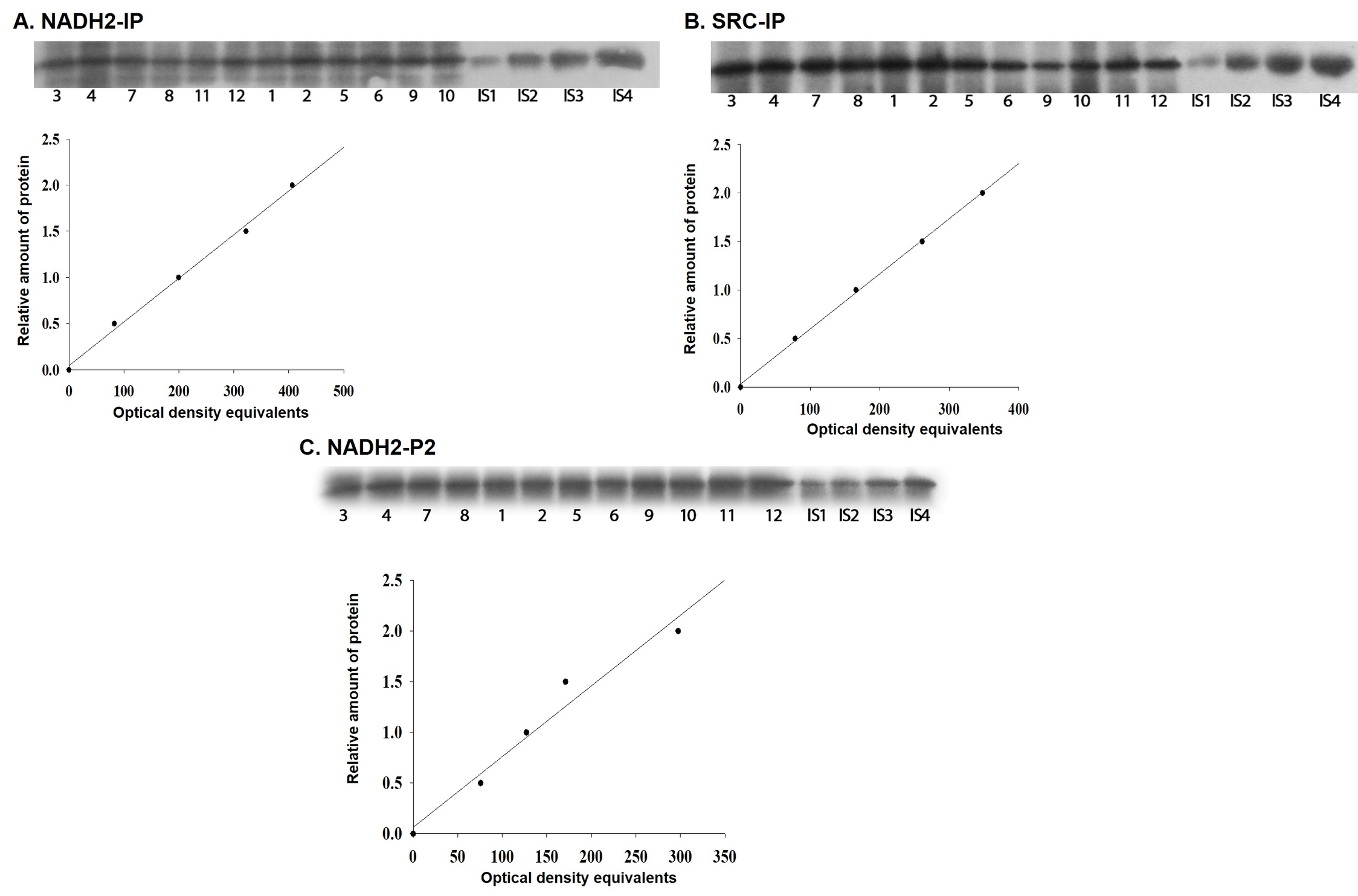

Supplement: S1 Fig — Sample films and calibration plots for: (A) NADH2-IP; (B) Src-IP and (C) NADH2-P2. On all sample films: 1-Left IMM, Good learner chick; 2-Right IMM Good learner chick; 3-Left PPN Good learner chick; 4- Right PPN Good Learner chick; 5-Left IMM, Poor learner chick; 6-Right IMM Poor learner chick; 7-Left PPN Poor learner chick; 8- Right PPN Poor Learner chick; 9-Left IMM, Untrained chick; 10-Right IMM Untrained chick; 11-Left PPN Untrained chick; 12- Right PPN Untrained chick. All sample gels contains internal standards–IS1, IS2, IS3,IS4 containing, respectively, 15, 30, 45, and 60 μg protein and corresponding to 0.5, 1.0, 1.5 and 2.0 relative amounts of protein respectively. For each calibration the r2 value was more than 97%. Note that the effects reported in this paper were obtained from the complete dataset and are not necessarily apparent from visual inspection of a single gel. (TIF) [file pone.0297166.s006.tif]

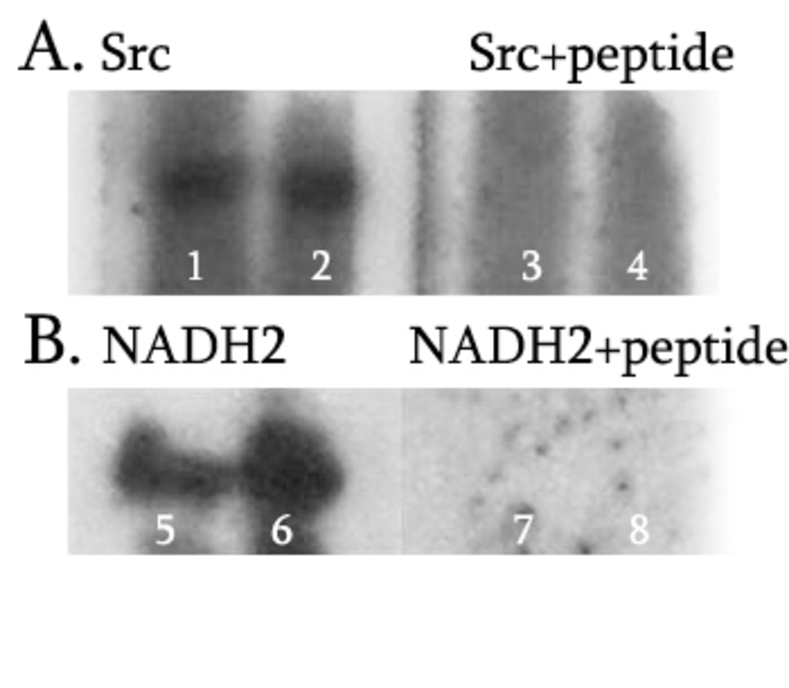

Supplement: S2 Fig — A-Western immunoblot image for Src staining. Lanes 1,2 –immunostaining with Src antibodies, Lanes 3,4 –immunostaining with Src antibodies containing corresponding antigen peptide. B- A-Western immunoblot image for NADH2 staining. Lanes 5,6 –immunostaining with NADH2 antibodies, Lanes 7,8 –immunostaining with NADH2 antibodies containing corresponding antigen peptide. (TIF) [file pone.0297166.s007.tif]

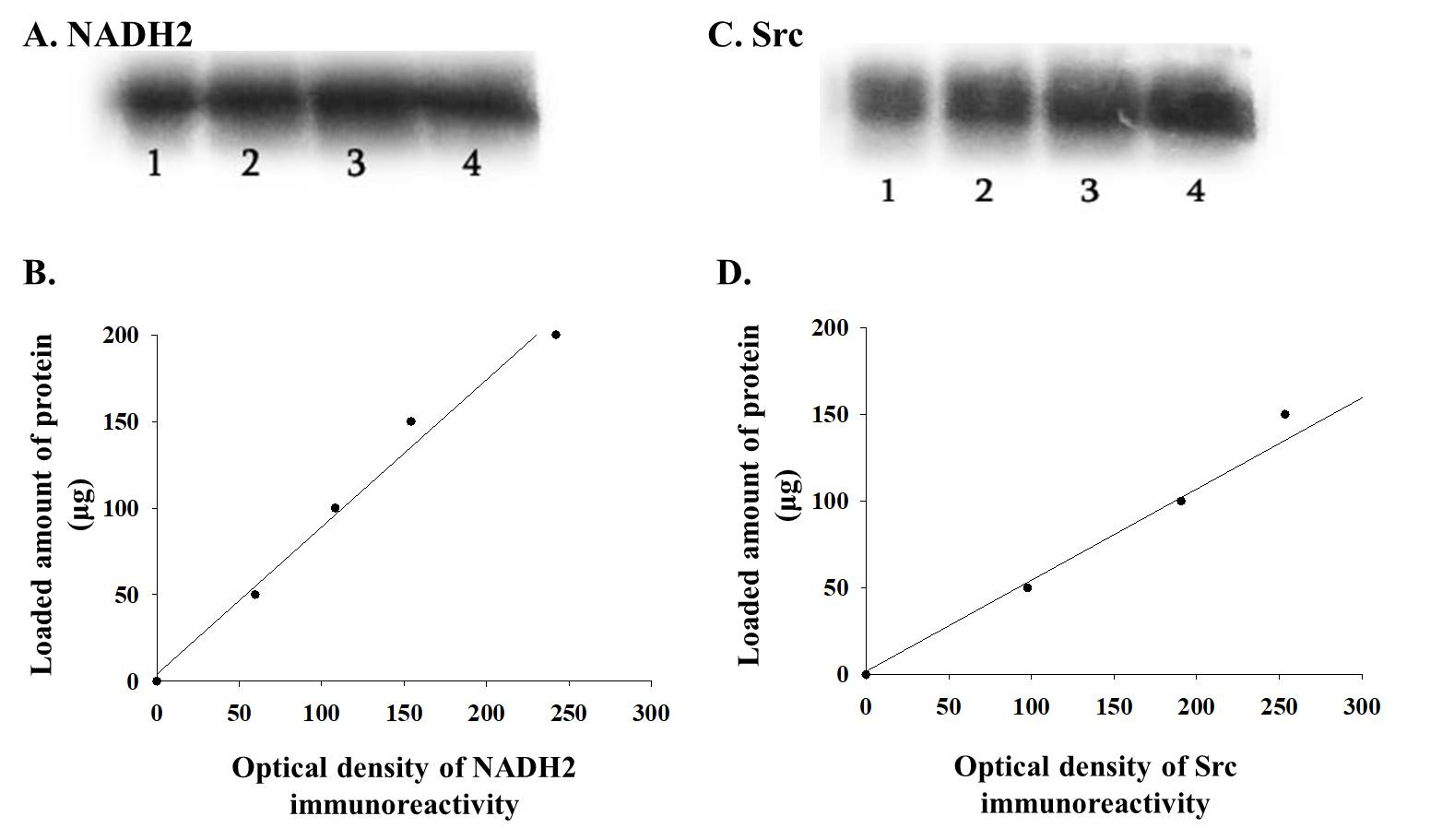

Supplement: S3 Fig — Four different amounts (50, 100, 150 and 200 μg of protein) of RIPA buffer-solubilized and Protein A/G agarose-precleared fractions were applied to Src antibodies immobilized on columns. The Western blots of eluates were stained with antibodies against Src and NADH2 proteins. A-NADH2 immunostaining; B-regression plot of NADH2 immunoreactivity with the amount of loaded protein on IA chromatography; C-Src immunostaining; D-regression plot of Src immunoreactivity with the amount of loaded protein on IA chromatography. For both Src and NADH2, least-squares regression showed a virtually perfect fit to a straight line. (TIF) [file pone.0297166.s008.tif]

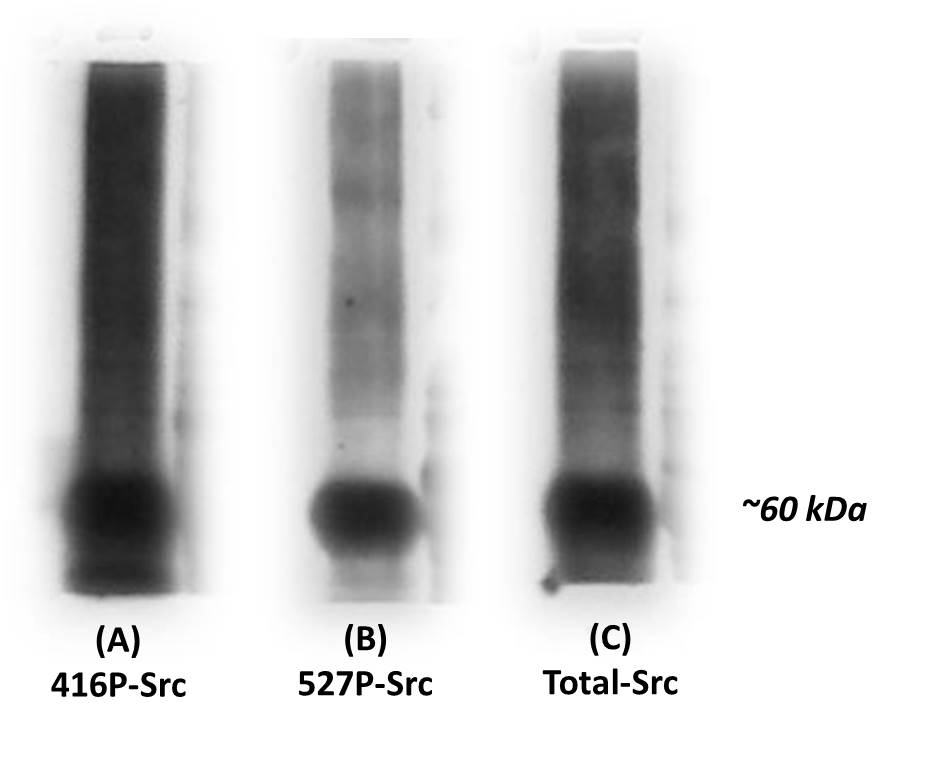

Supplement: S4 Fig — The blots were stained with antibodies against: 416P-Src (A), 527P-Src (B) and total Src (C). Both phosphorylated forms of Src are interacting with NADH2. (TIF) [file pone.0297166.s009.tif]

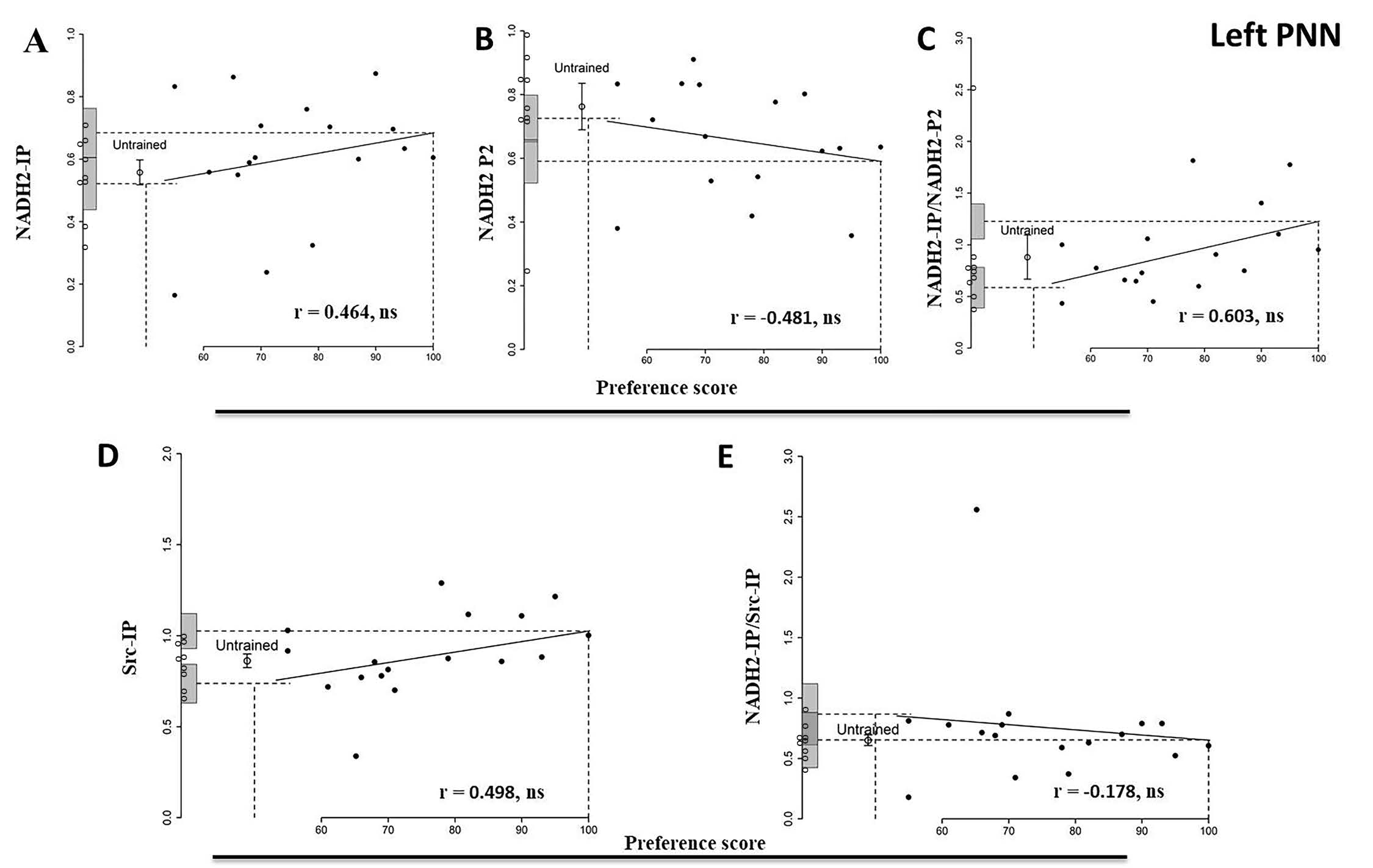

Supplement: S5 Fig — Preference score plotted against standardized relative amounts of NADH2-IP (A), NADH2-P2 (B), NADH2-IP/NADH2-P2 (C), Src-IP (D) and NADH2-IP/SRC-IP (E).Conventions otherwise as for Fig 1. No correlation was significant. (TIF) [file pone.0297166.s010.tif]

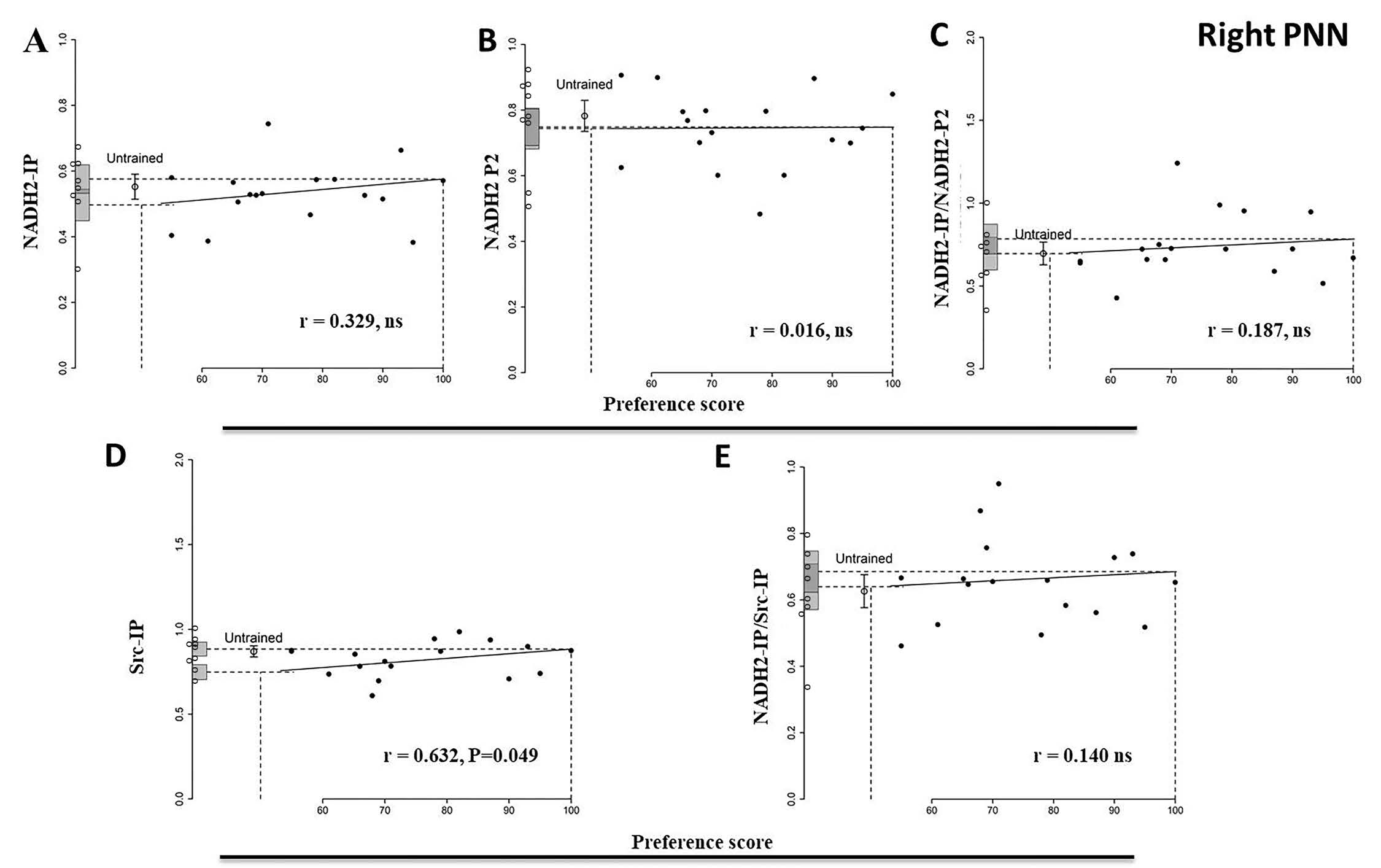

Supplement: S6 Fig — Preference score plotted against standardized relative amounts of NADH2-IP (A), NADH2-P2 (B), NADH2-IP/NADH2-P2 (C), Src-IP (D) and NADH2-IP/SRC-IP (E). Conventions otherwise as for Fig 1. No correlation was significant. (TIF) [file pone.0297166.s011.tif]

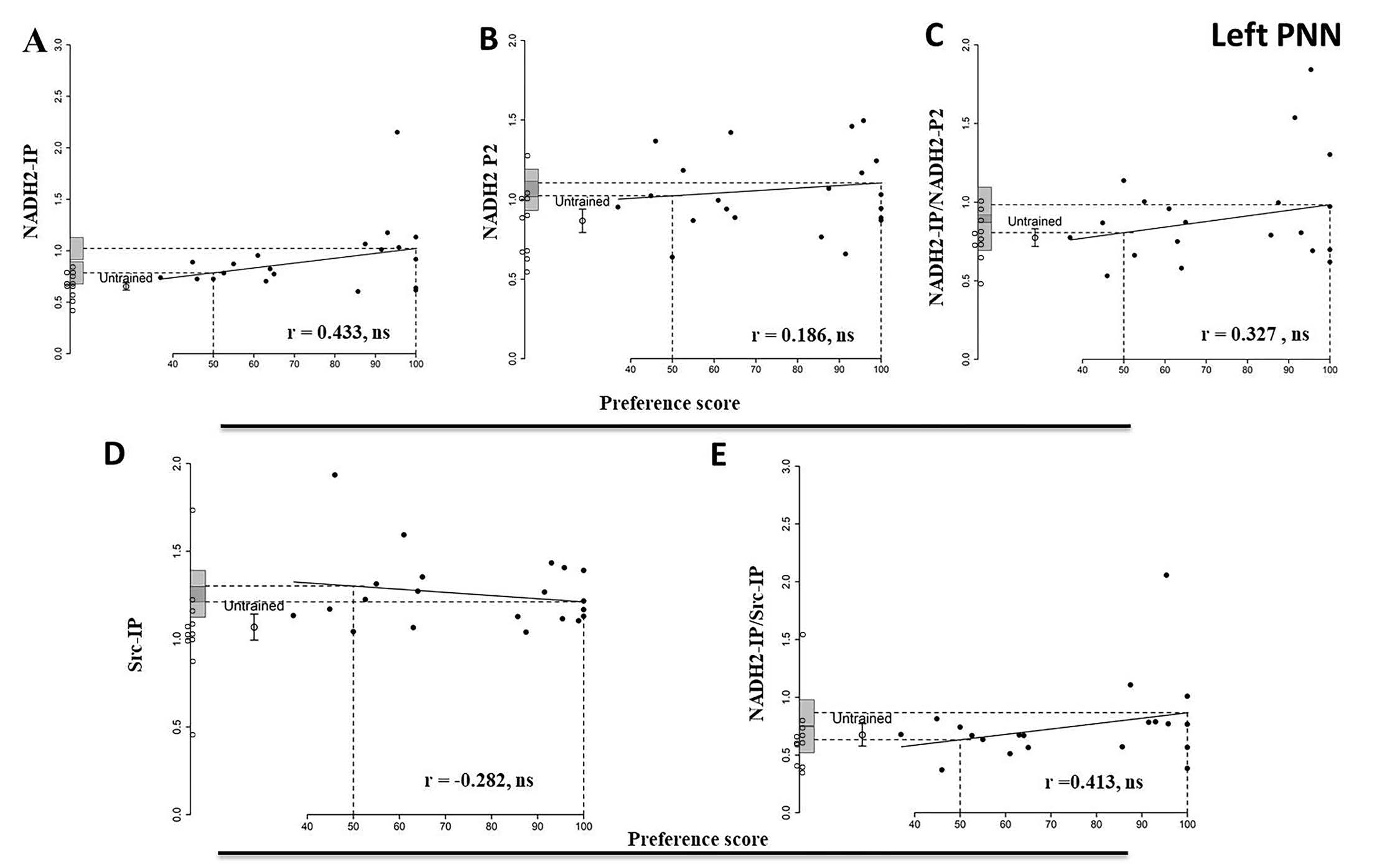

Supplement: S7 Fig — Preference score plotted against standardized relative amounts of NADH2-IP (A), NADH2-P2 (B), NADH2-IP/NADH2-P2 (C), Src-IP (D) and NADH2-IP/SRC-IP (E).Conventions otherwise as for Fig 1. No correlation was significant. (TIF) [file pone.0297166.s012.tif]

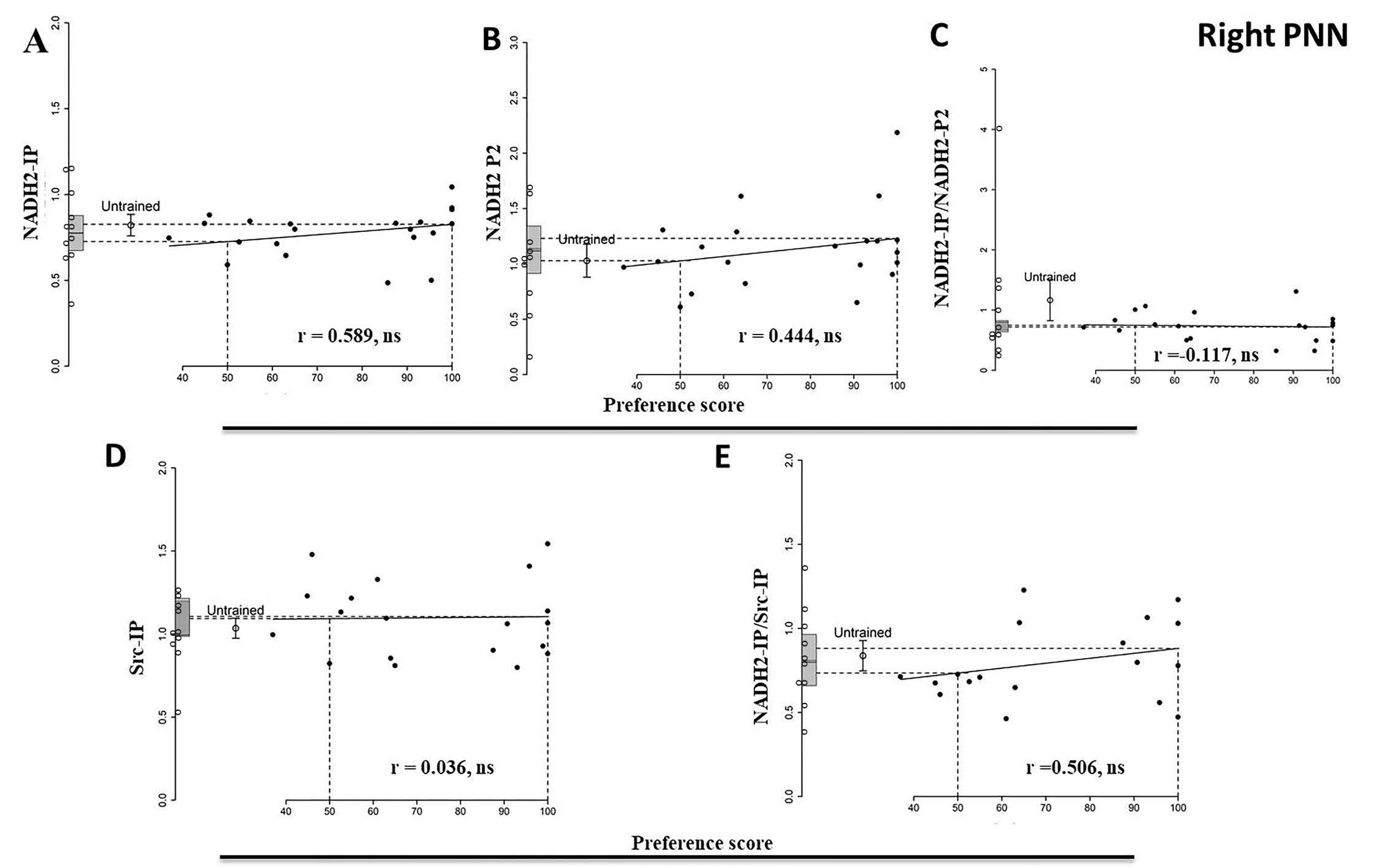

Supplement: S8 Fig — Preference score plotted against standardized relative amounts of NADH2-IP (A), NADH2-P2 (B), NADH2-IP/NADH2-P2 (C), Src-IP (D) and NADH2-IP/SRC-IP (E). Conventions otherwise as for Fig 1. No correlation was significant. (TIF) [file pone.0297166.s013.tif]
