# Supplementary material for: Src-NADH dehydrogenase subunit 2 complex and recognition memory of imprinting in domestic chicks
Source: PLoS One. 2024 Jan 29;19(1):e0297166. doi: 10.1371/journal.pone.0297166 (PMC10824410; doi:10.1371/journal.pone.0297166)
Supplement: S1 Raw images — (PDF) [file pone.0297166.s014.pdf]

Images of raw blots

The nitrocellulose membranes were cut into two parts as two antigens were stained on each of them (see manuscript- "Materials and Methods"). Molecular weight markers were stained with Ponceau S, and migration distances recorded. The band of Src corresponds to 55 KDa and NADH2 to 39 KDa. Optical densities of bands, marked with arrows, were measured.

Images of Src Immunoblots: Src-IP-1h after training

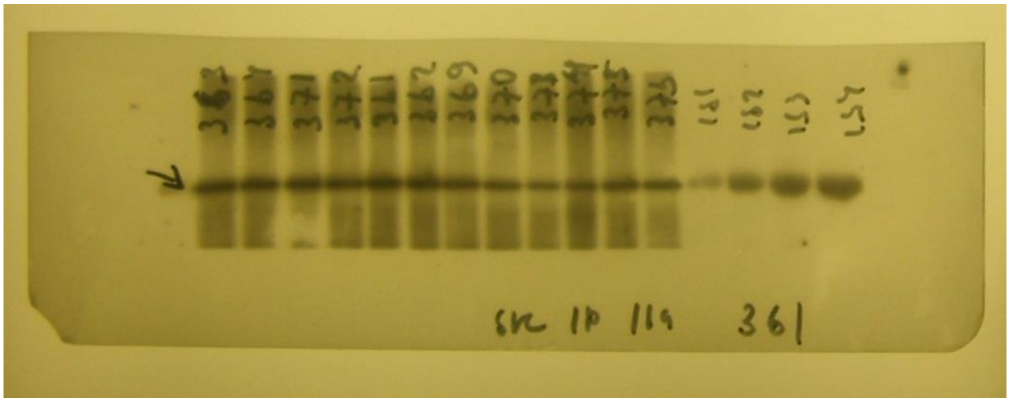

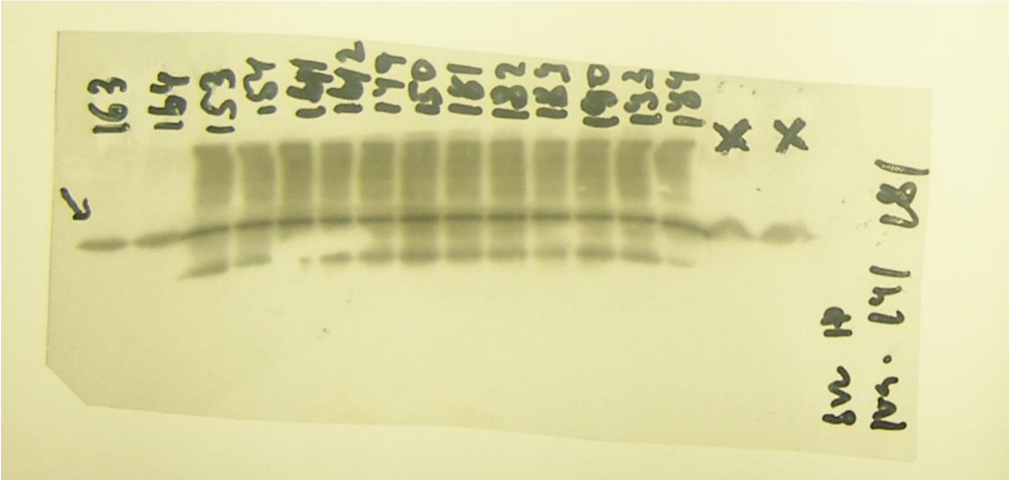

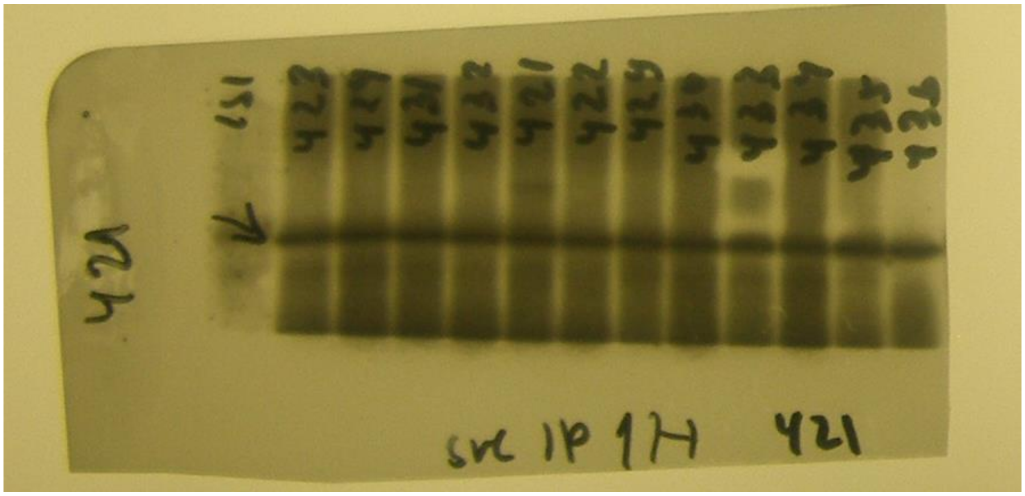
src IP 1H 421

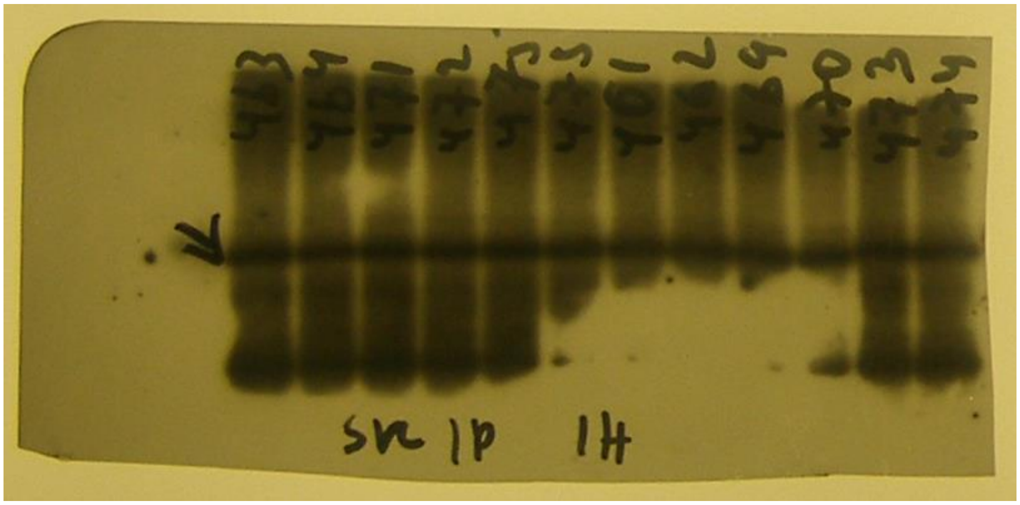
src IP 1H

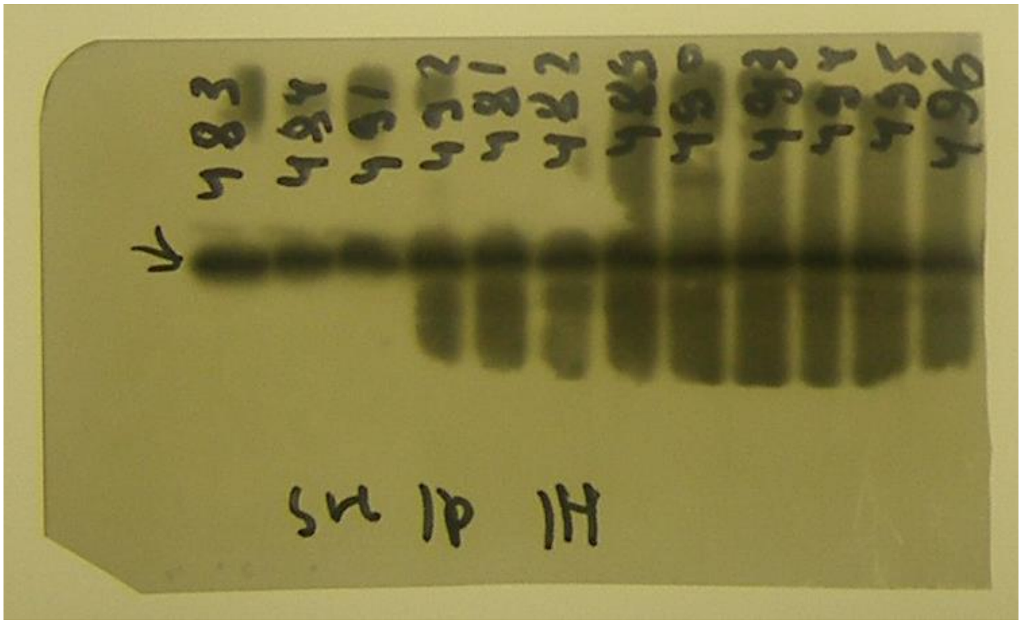
src IP 1H

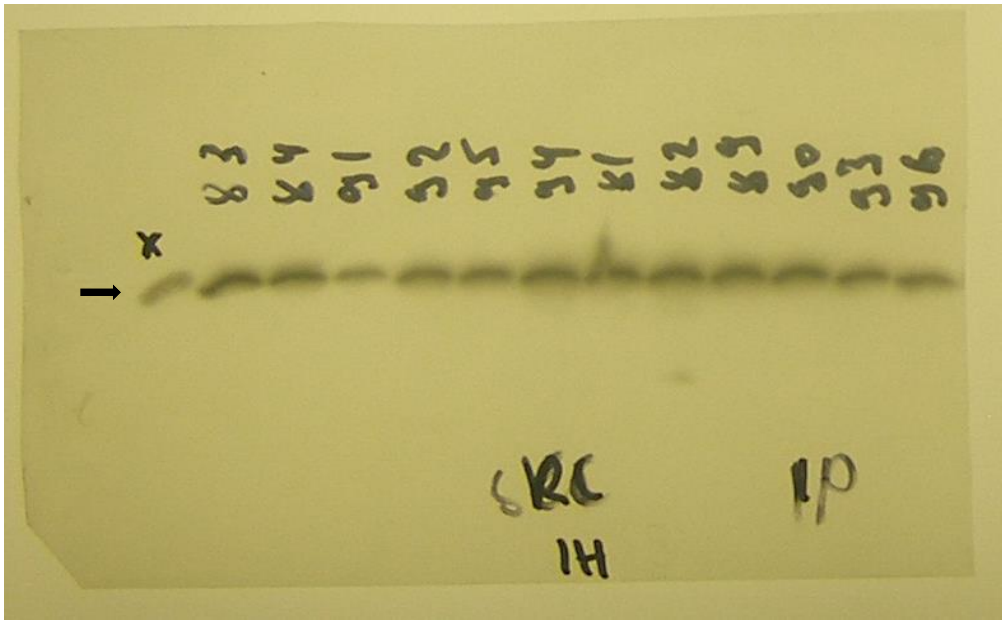
SRC IP
IH

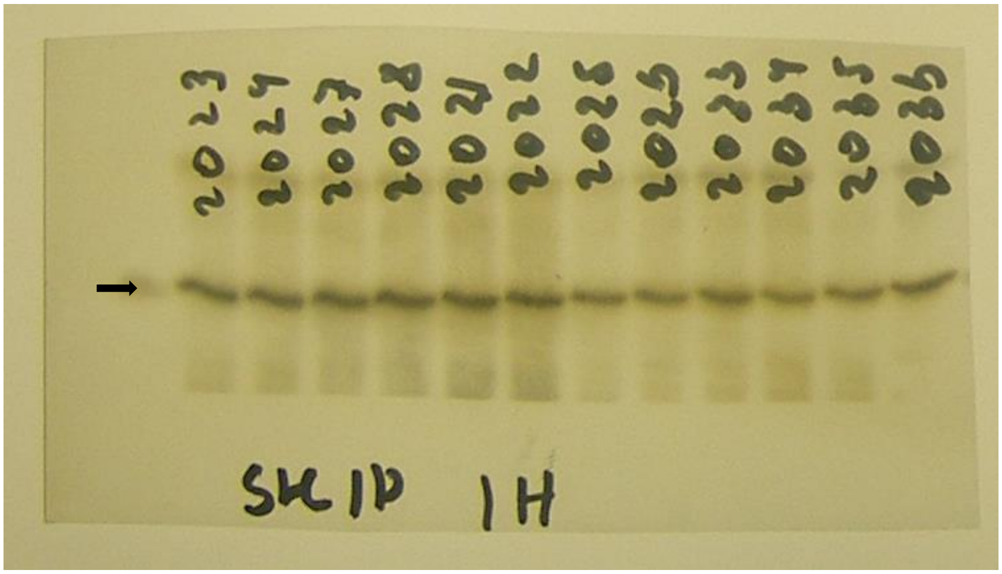
SK IP IH

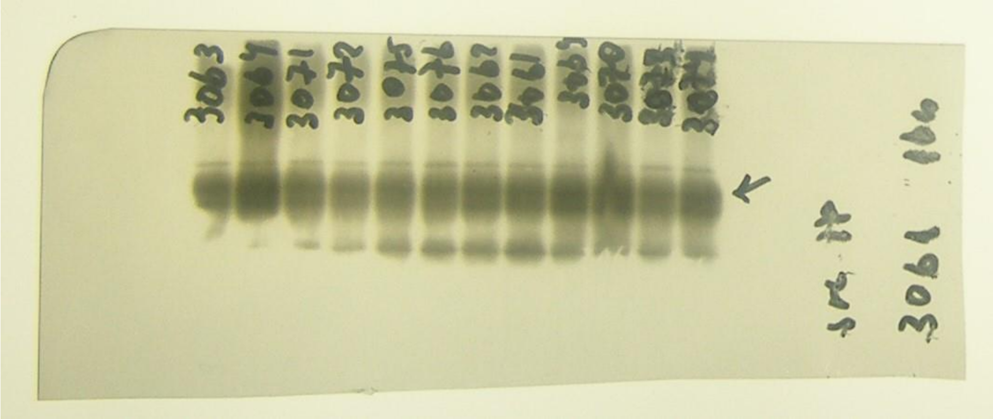

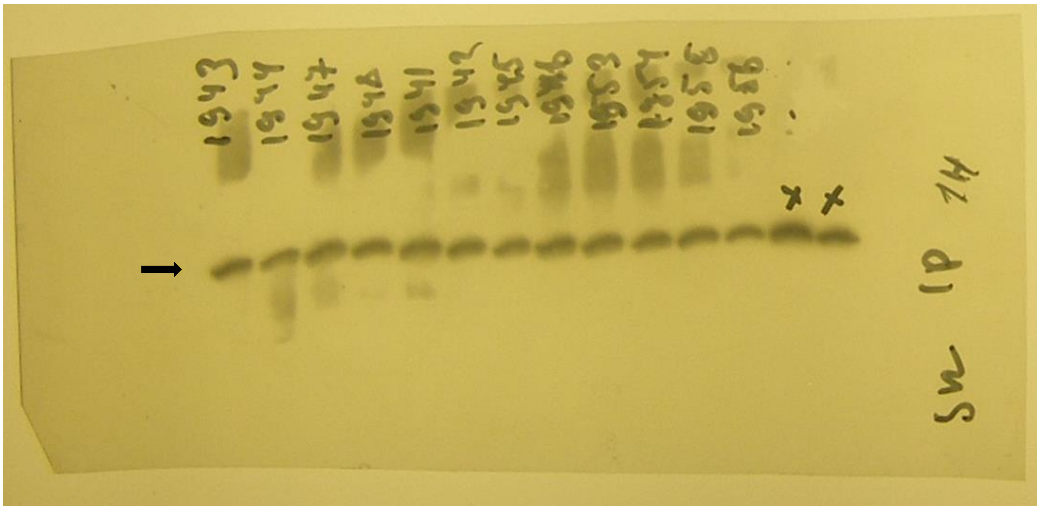

Images of NADH2 Immunoblots: NADH2-IP-1h after training

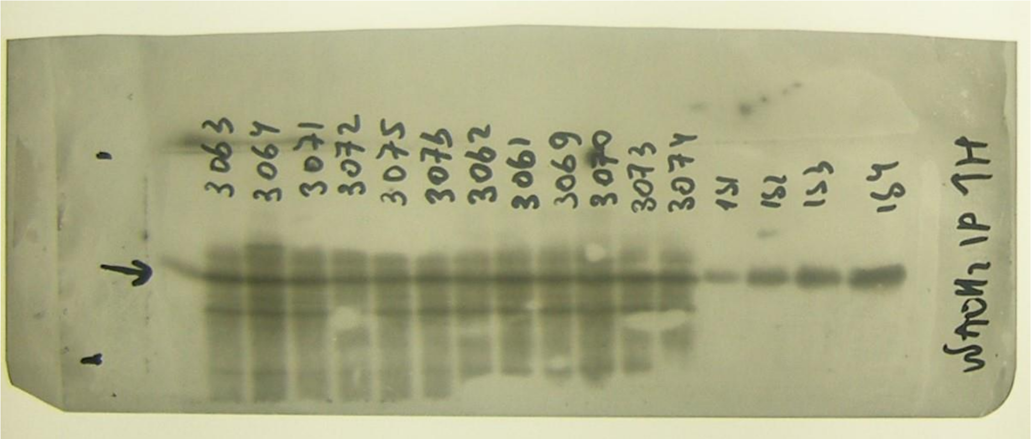

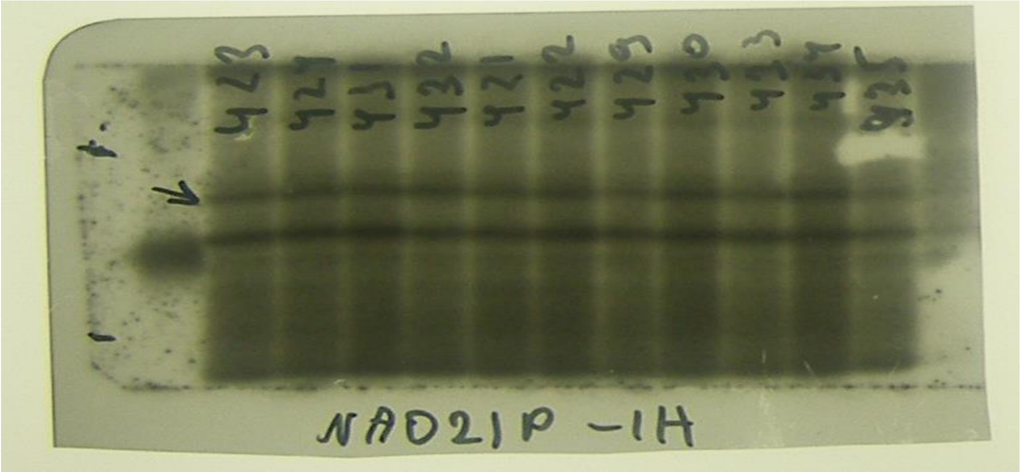

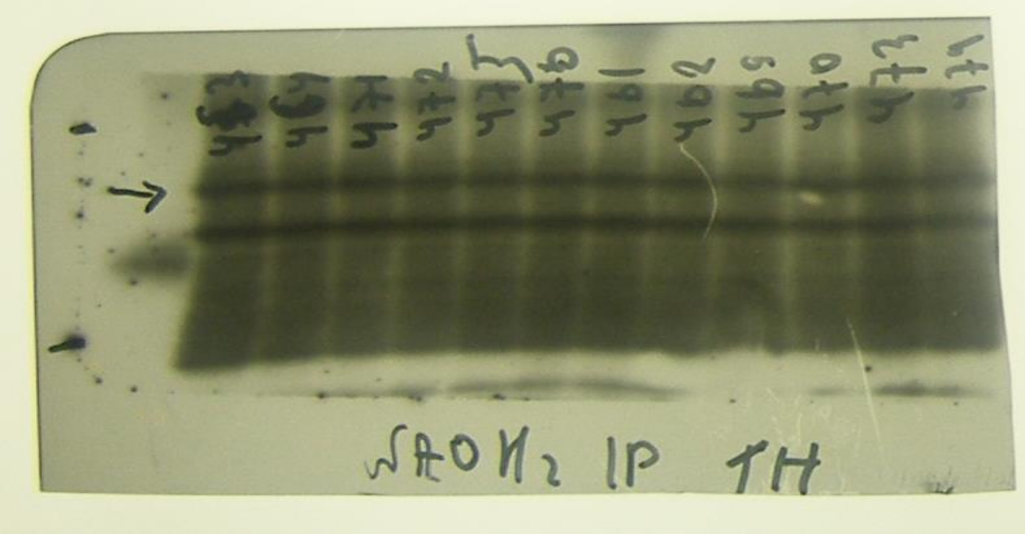

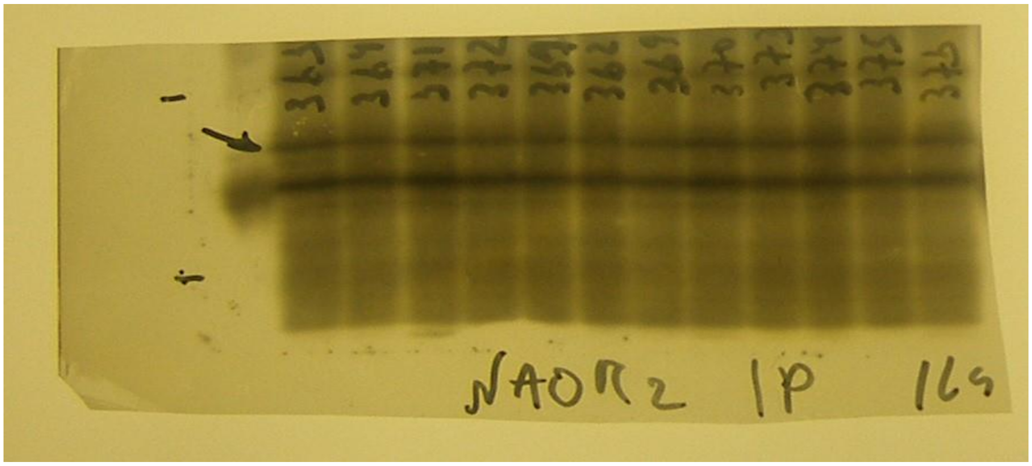
NAOR2 IP

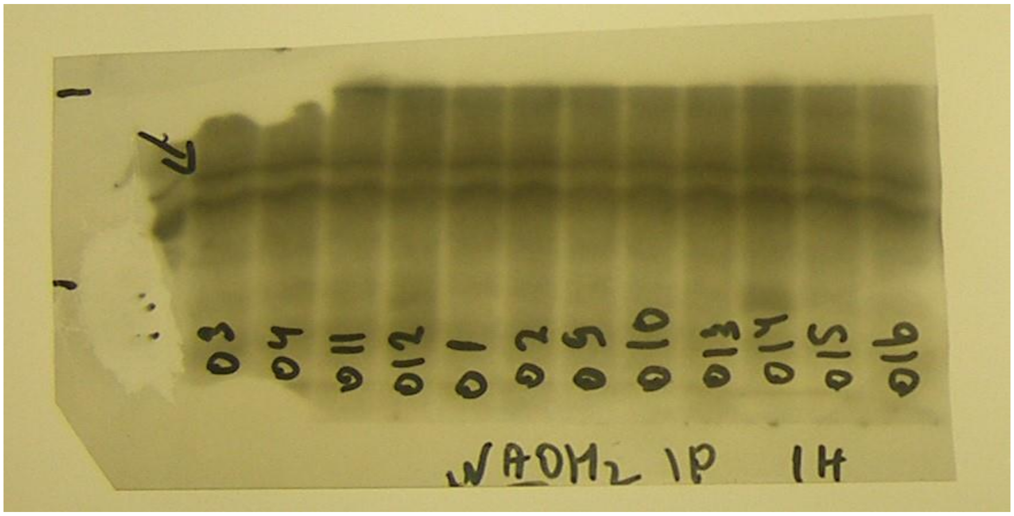
03 04 011 012 01 02 05 010 013 014 015 016
NADM2 IP 1H

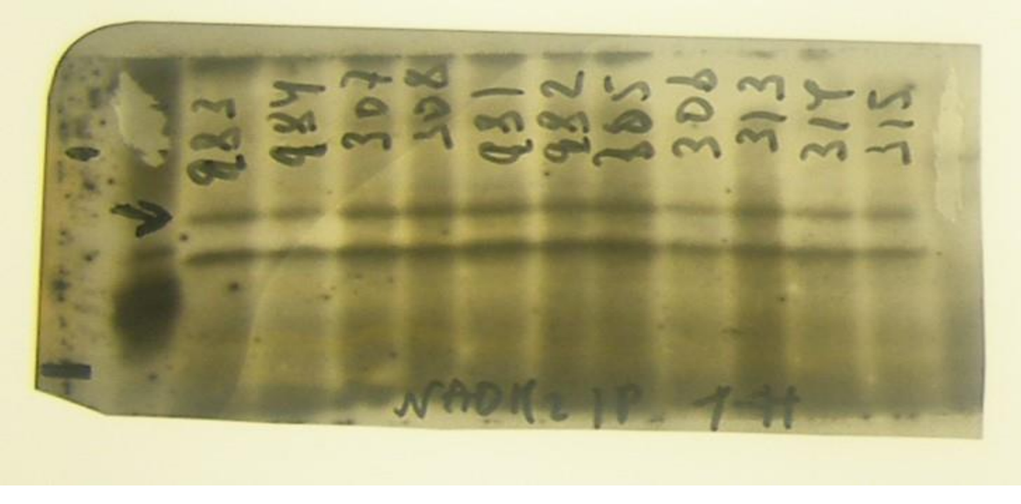
NADM2 IP

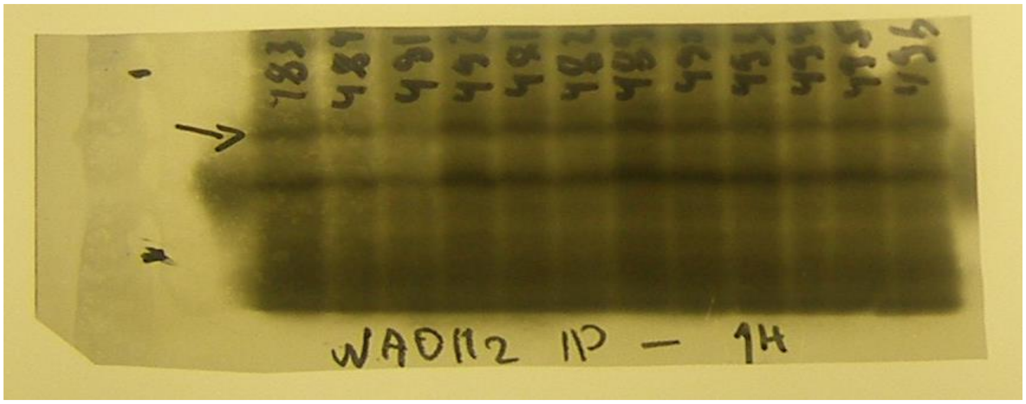

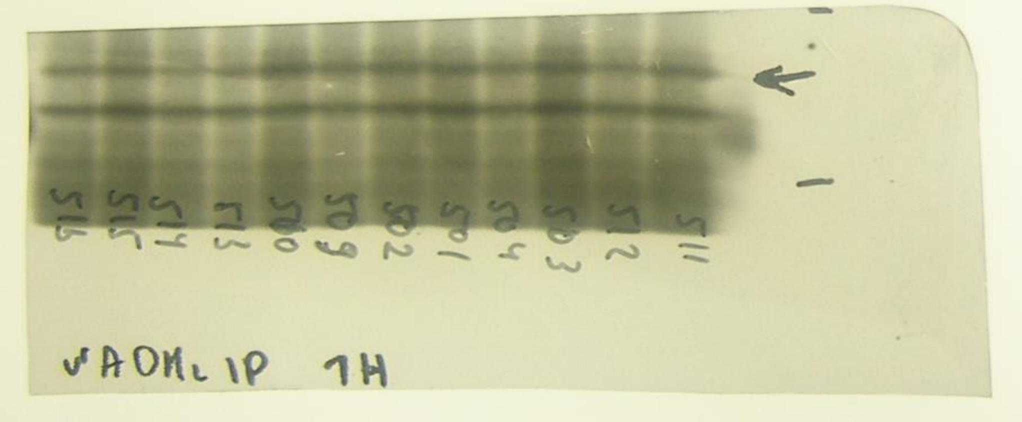

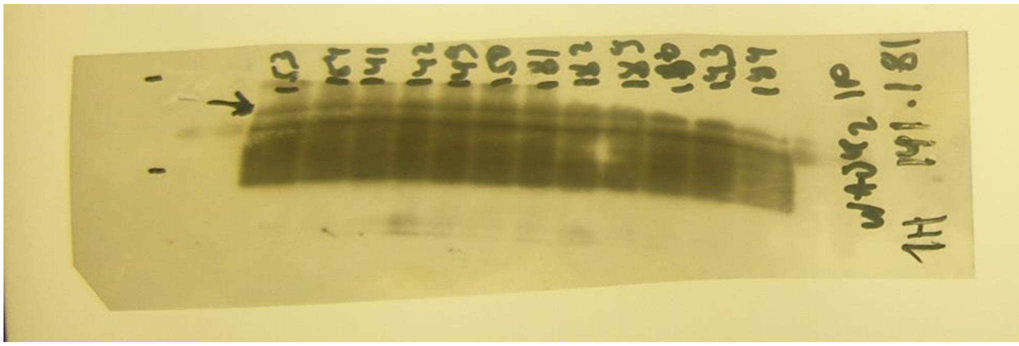

Images of NADH2 Immunoblots: NADH2-P2-1h after training

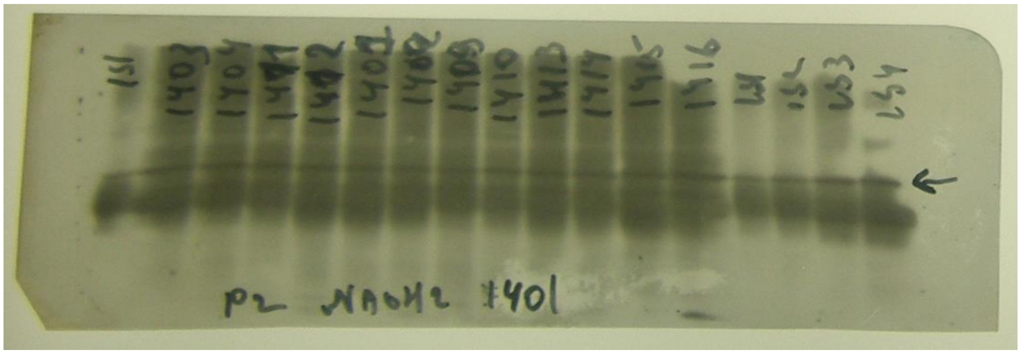

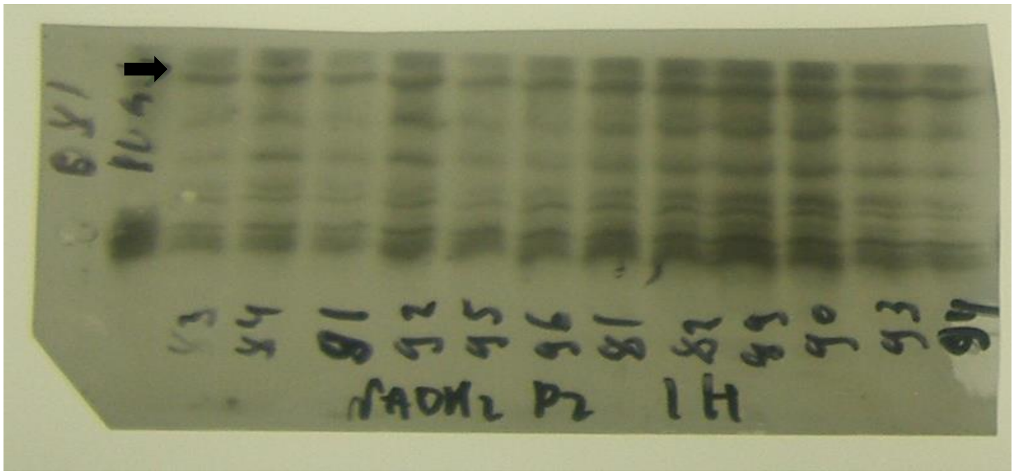

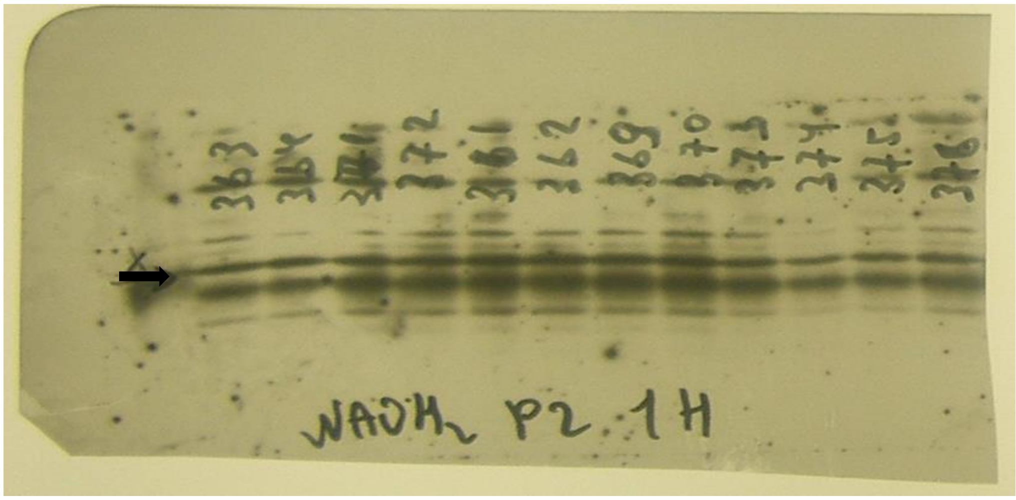

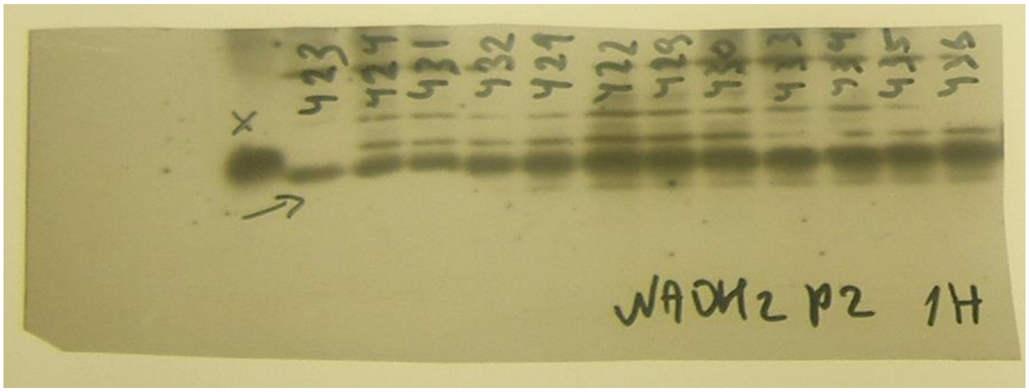
WADH2 P2 1H

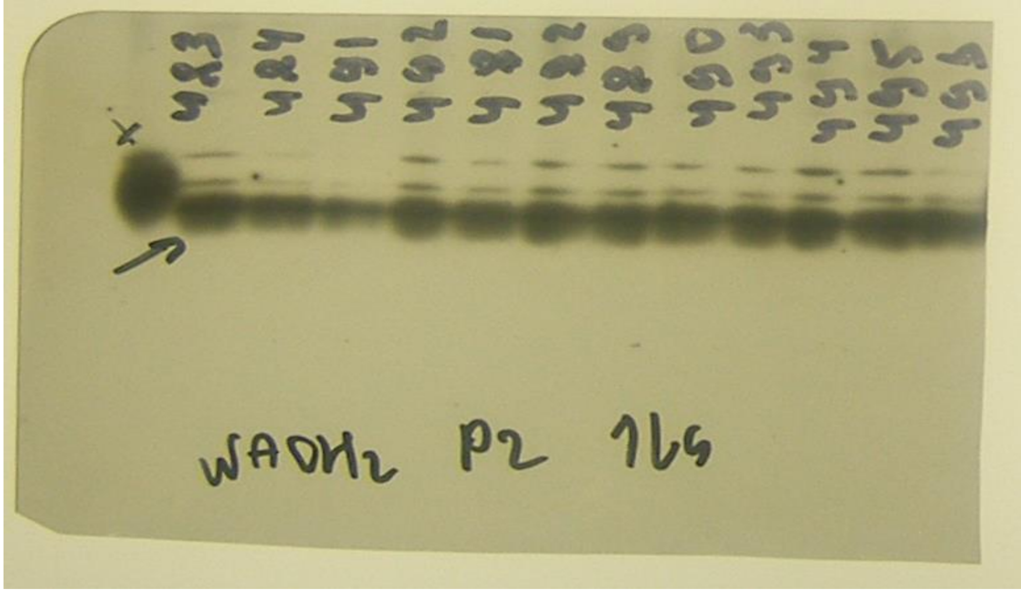
WADH2 P2

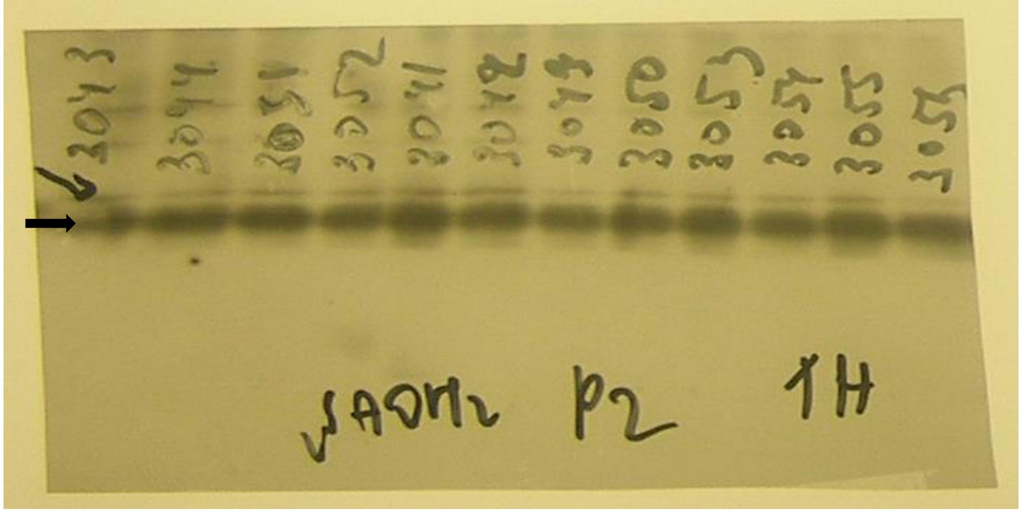
WADH2 P2 1H

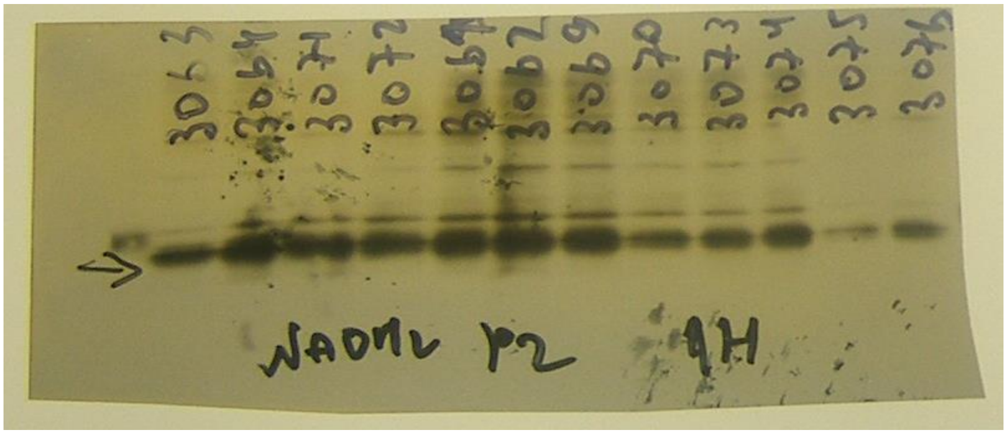

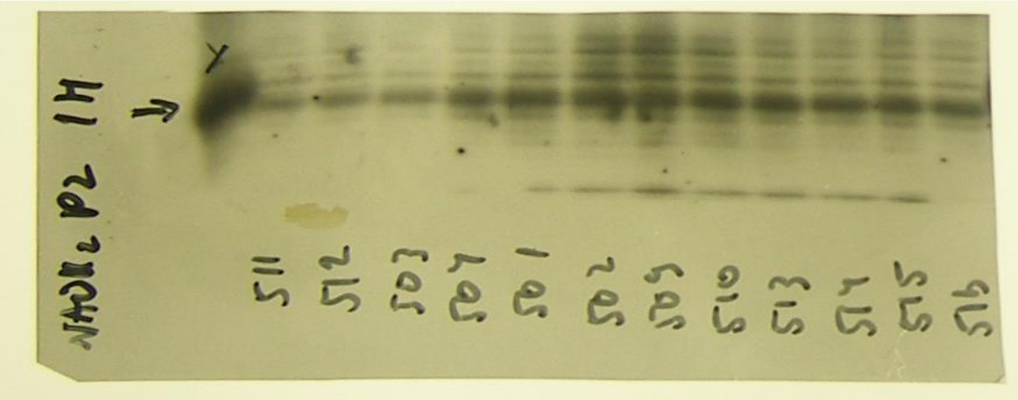

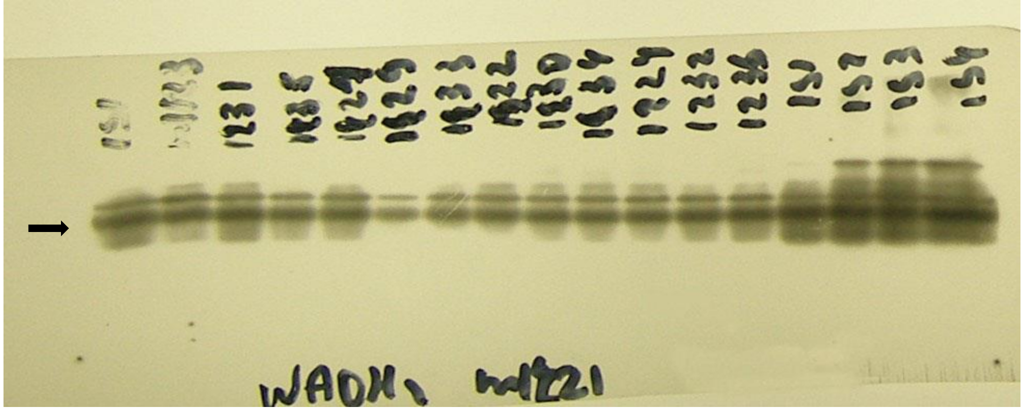

Images of Src Immunoblots: Src-IP-24h after training

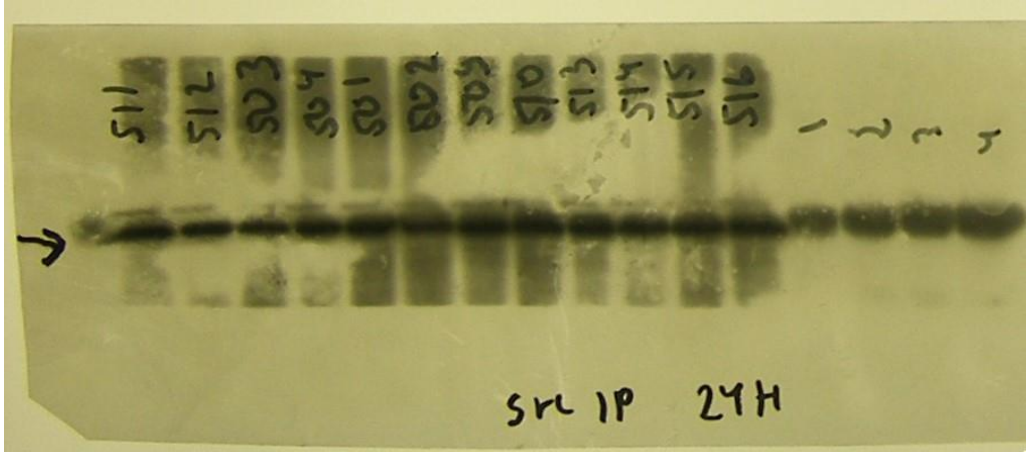

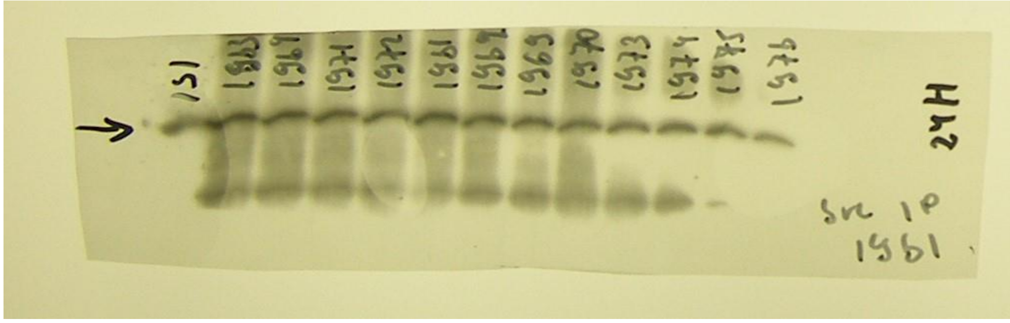

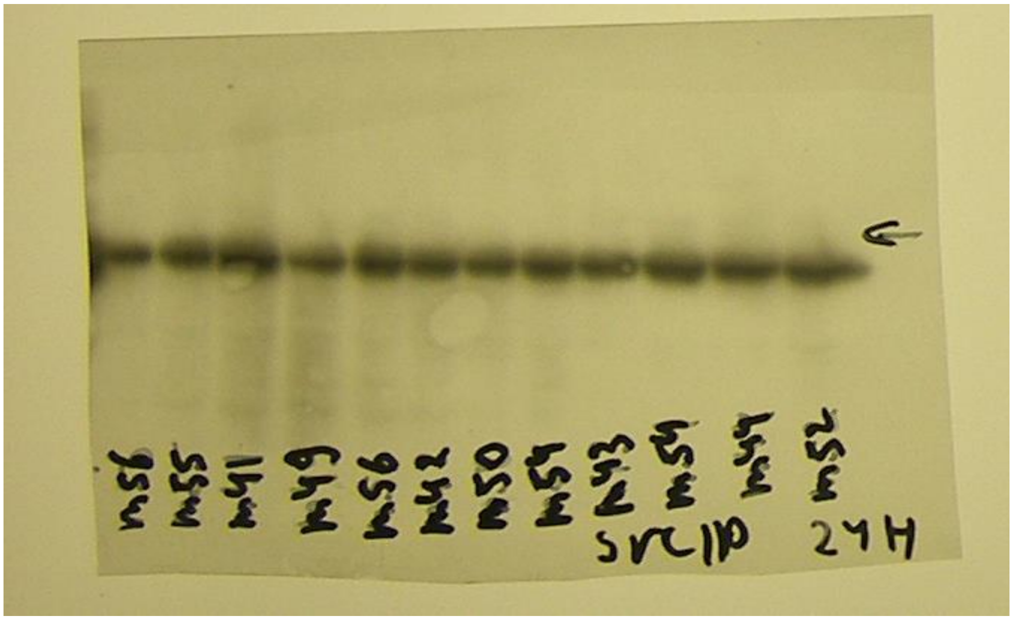

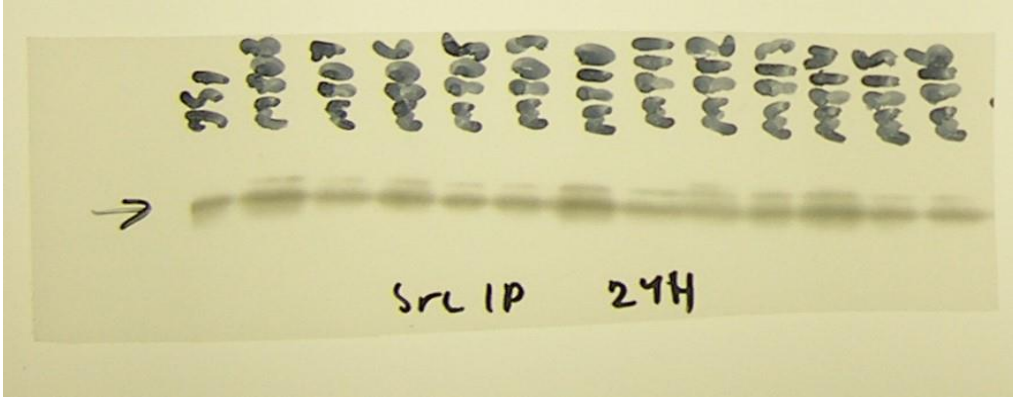
Src IP 24H

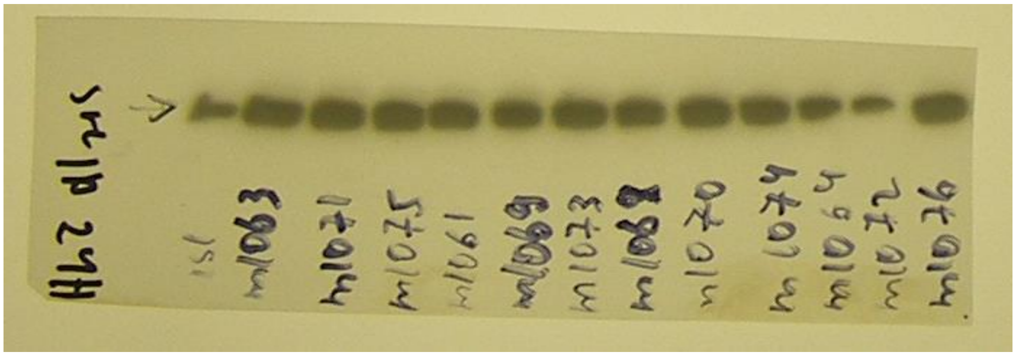
Src IP 24H

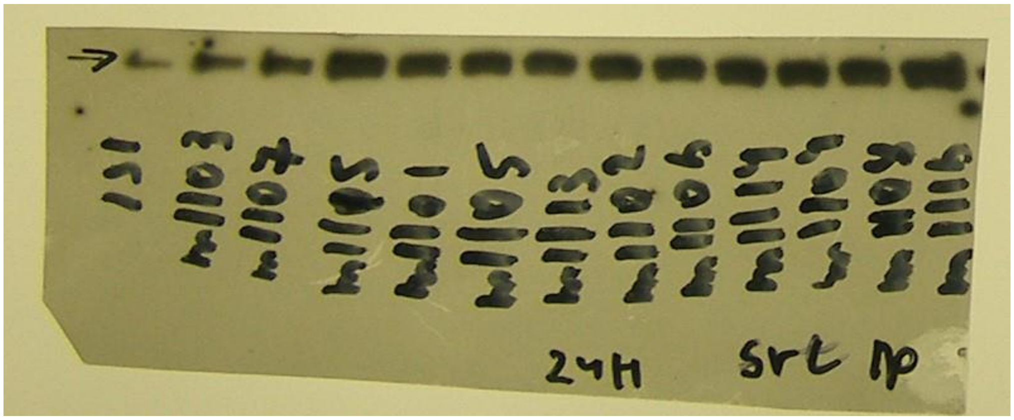
24H Src IP

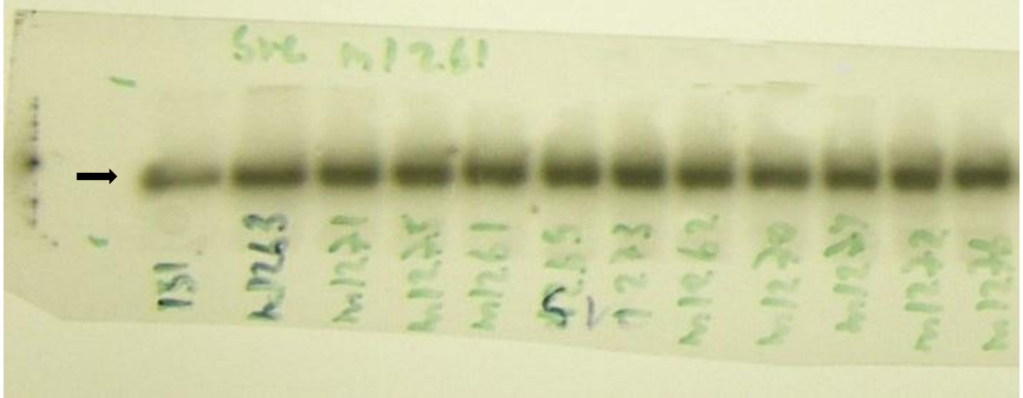
Src m1261

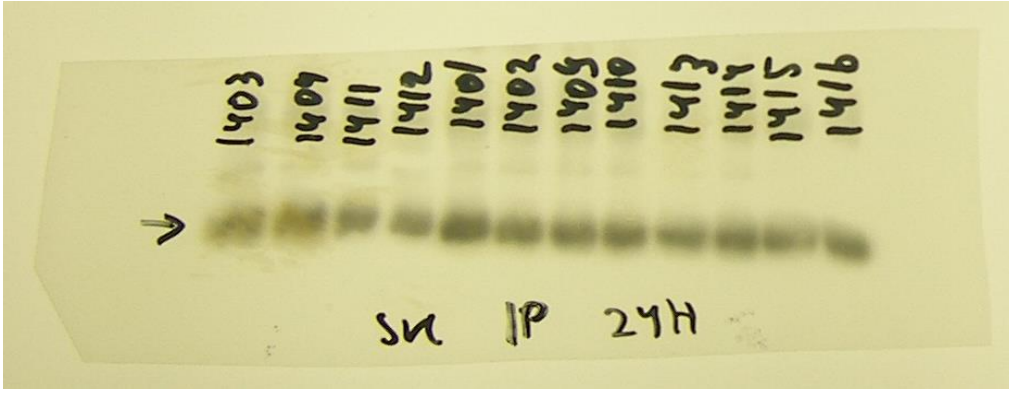
1403
1409
1411
1412
1401
1402
1405
1410
1413
1414
1415
1416
Src IP 24H

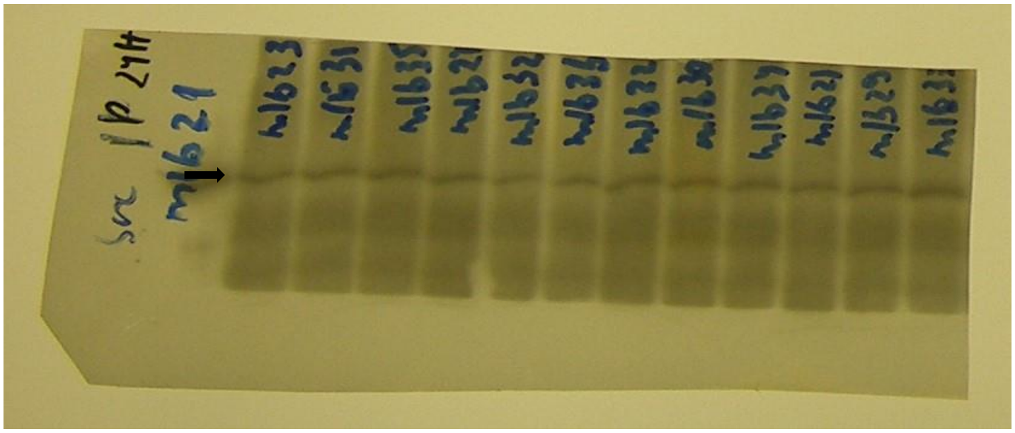
Src IP 24H

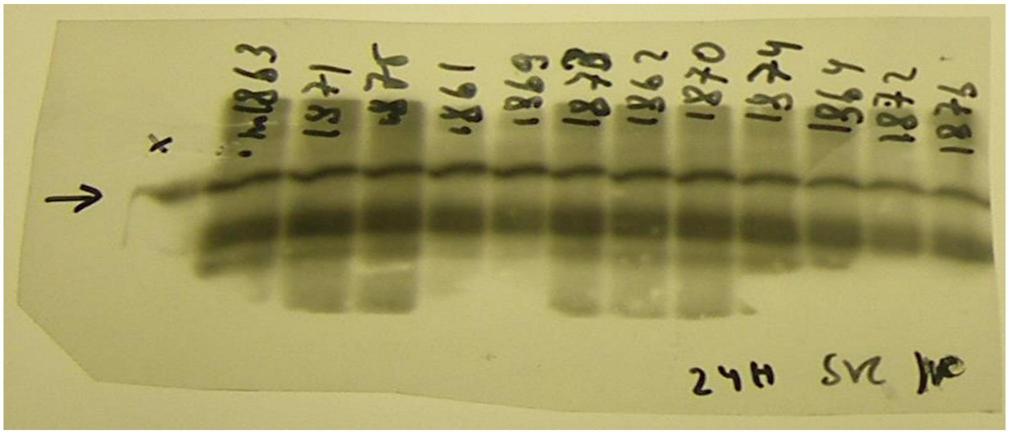
24H Src IP

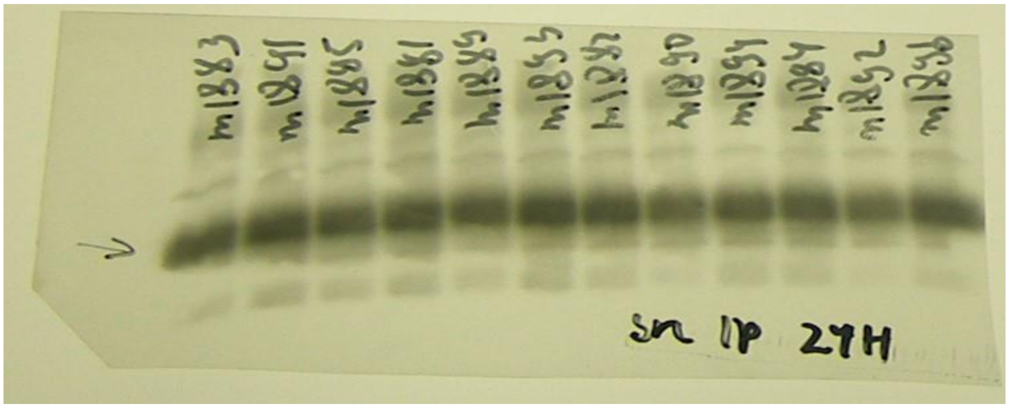
Src IP 24H

Images of NADH2 Immunoblots: NADH2-IP-24h after training

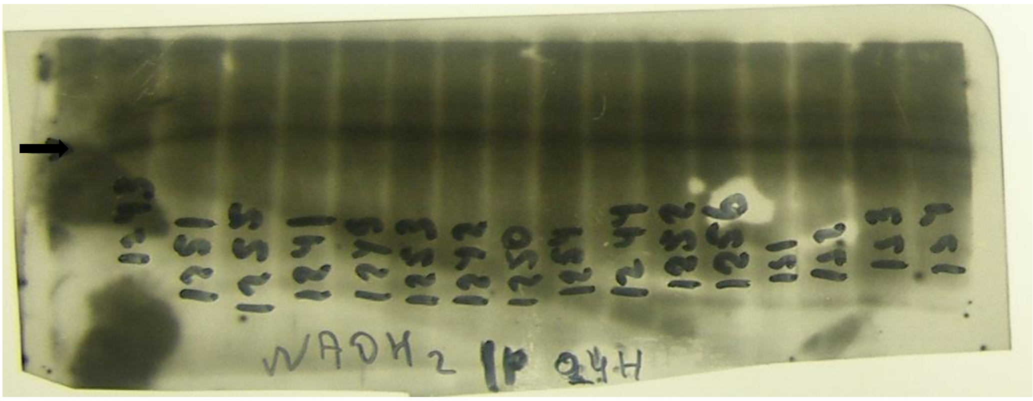

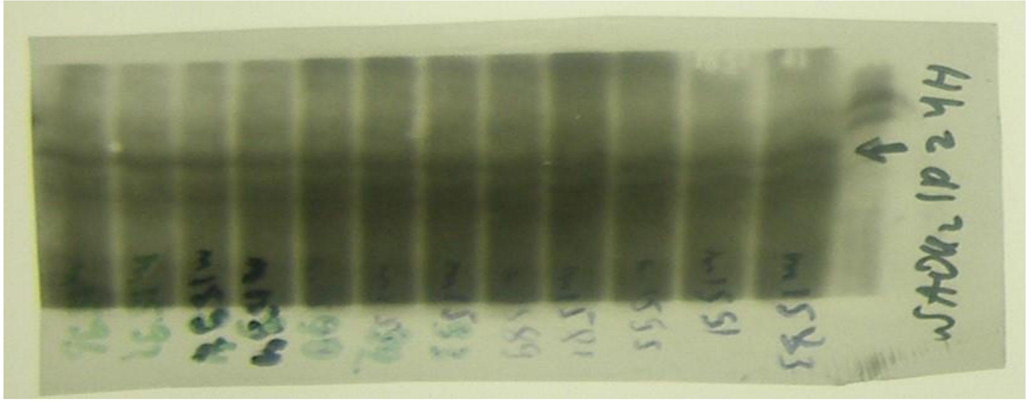

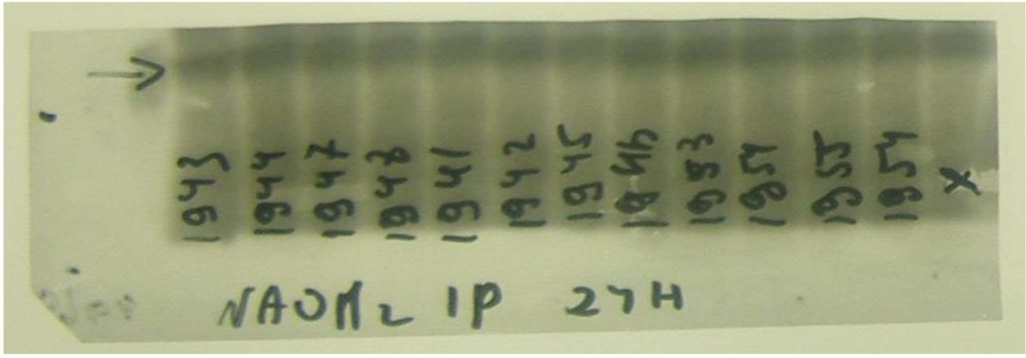

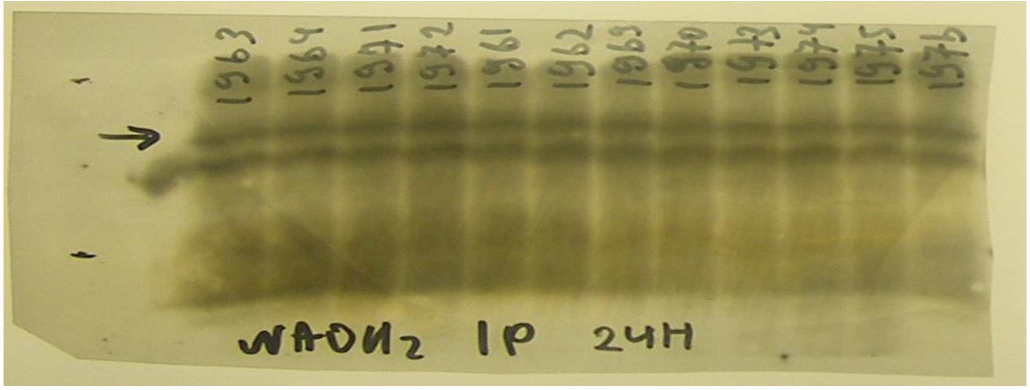
1963 1964 1971 1972 1961 1962 1969 1970 1973 1974 1975 1976
WADH2 IP 24H

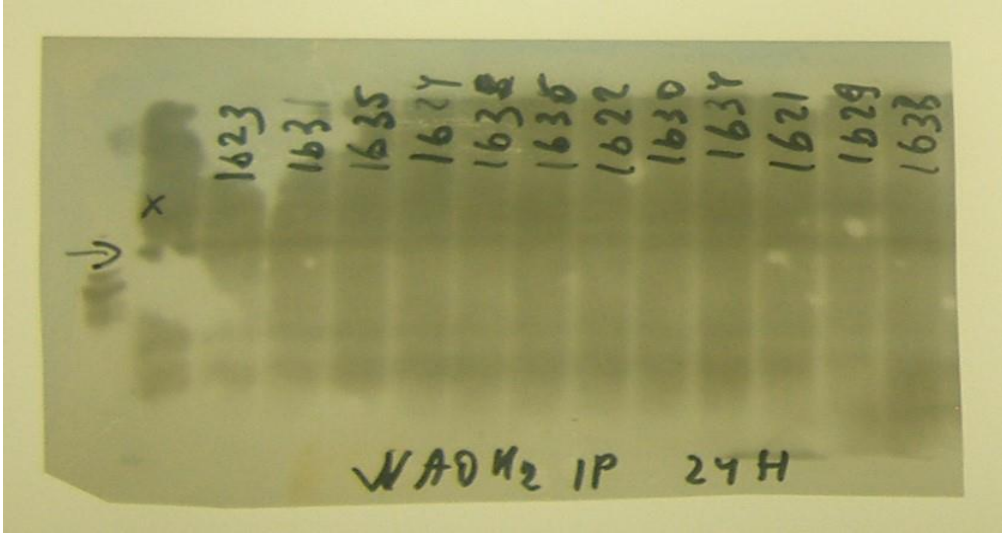
1623 1631 1635 1624 1636 1622 1630 1634 1621 1629
WADH2 IP 24H

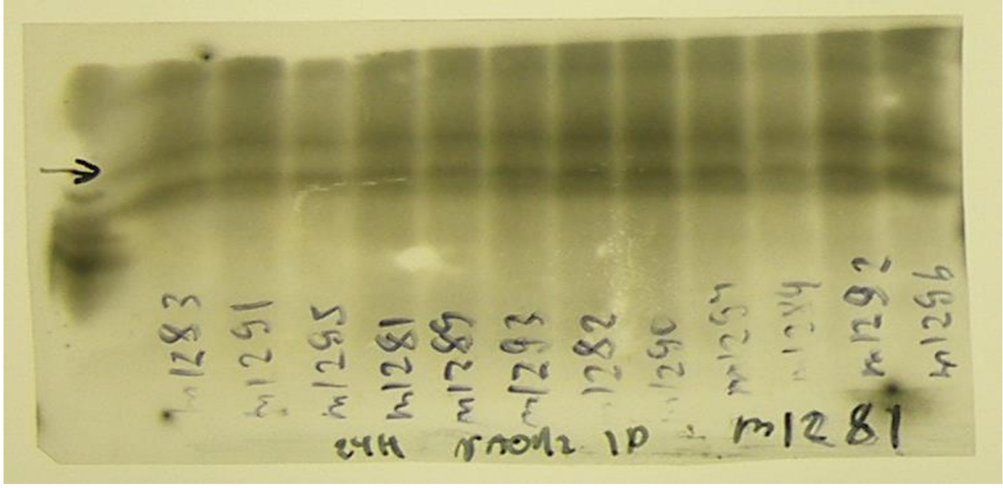
m1283 m1291 m1295 m1281 m1289 m1293 1282 1290 m1294 m1292 m1296
24H NADH2 IP : m1281

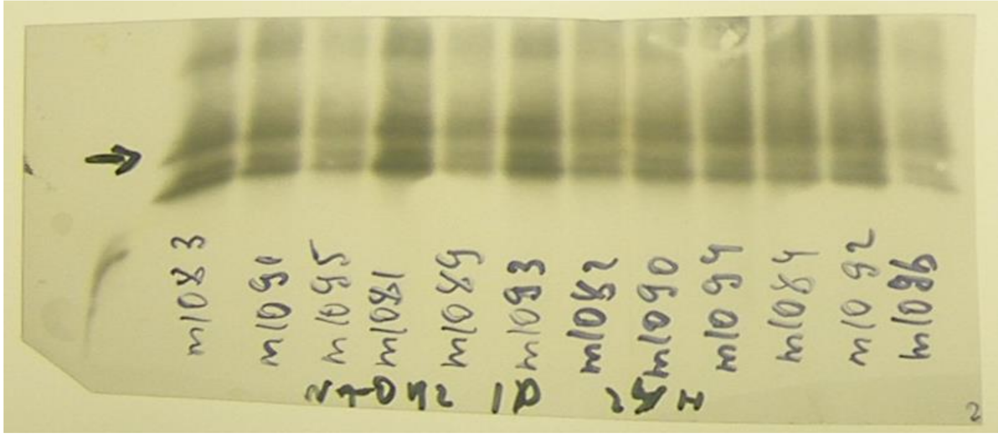
m1083
m1091
m1095
m1081
m1089
m1093
m1082
m1090
m1094
m1084
m1092
m1086

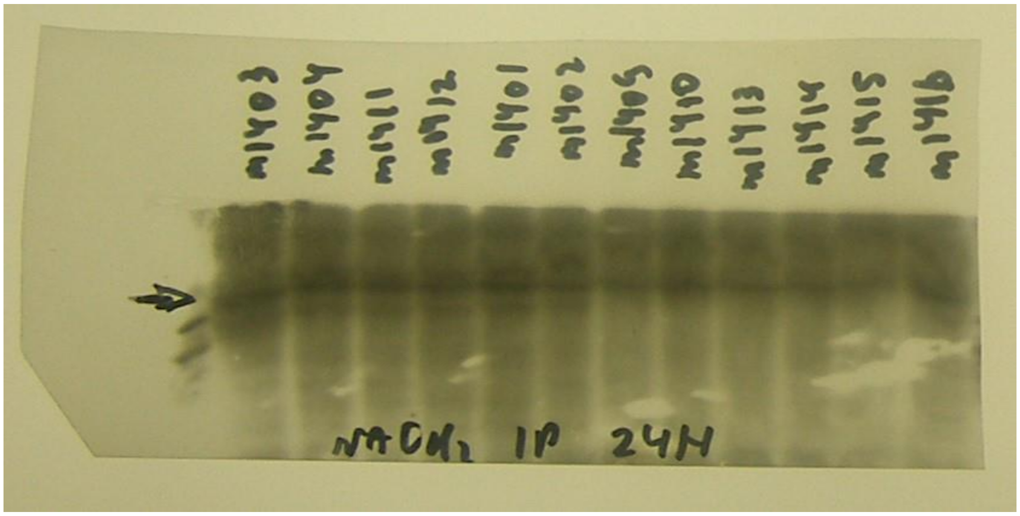
m1403
m1404
m1411
m1412
m1401
m1402
m1405
m1410
m1413
m1414
m1415
m1416

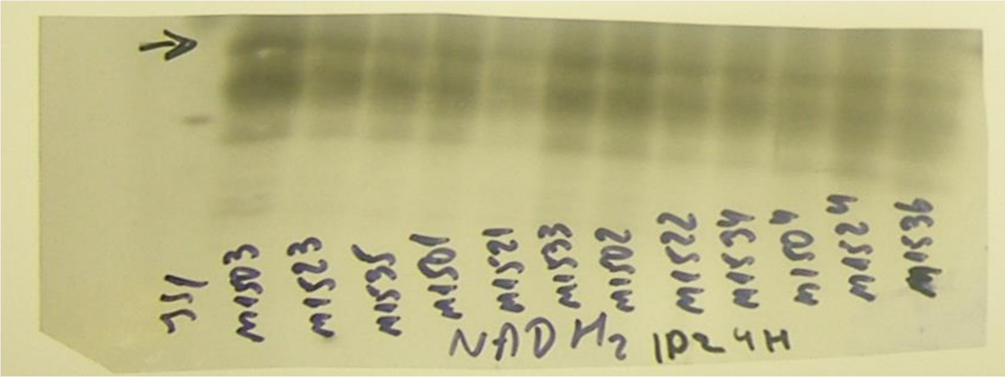
IP 24H

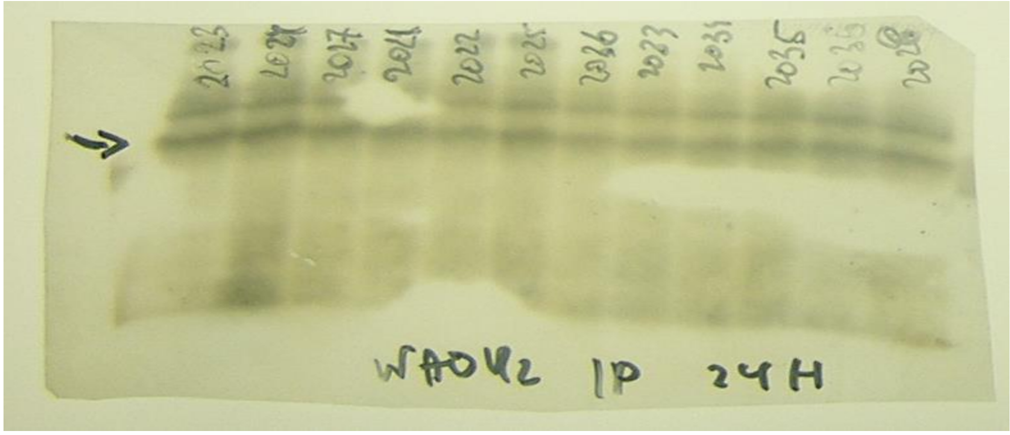

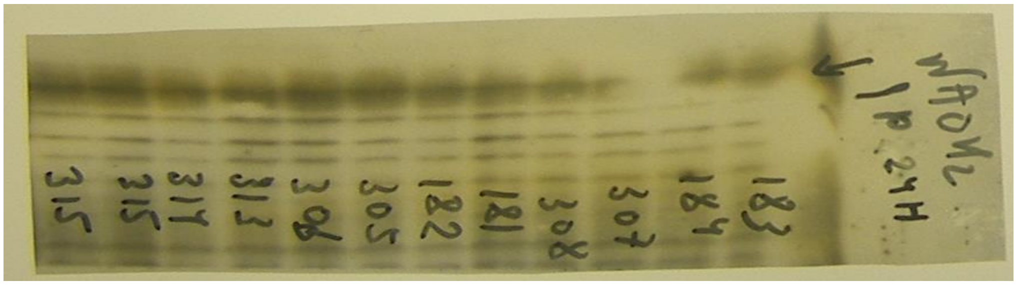

Images of NADH2 Immunoblots: NADH2-P2-24h after training

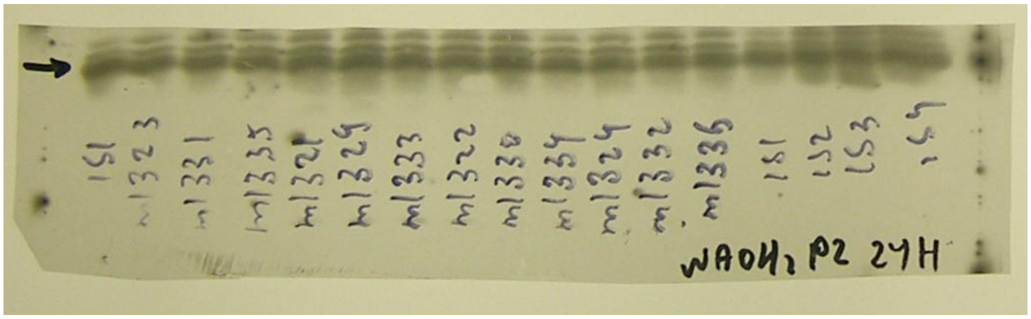

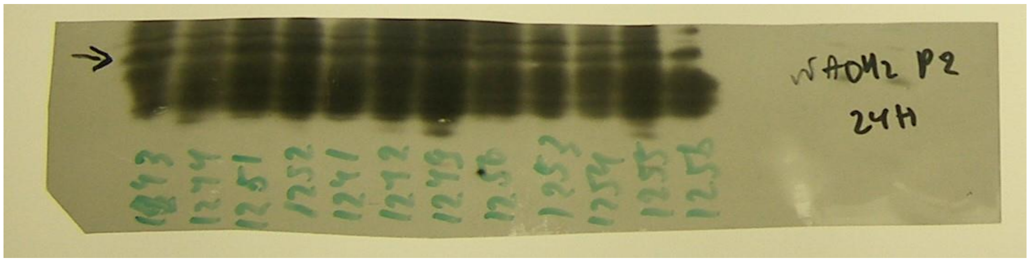

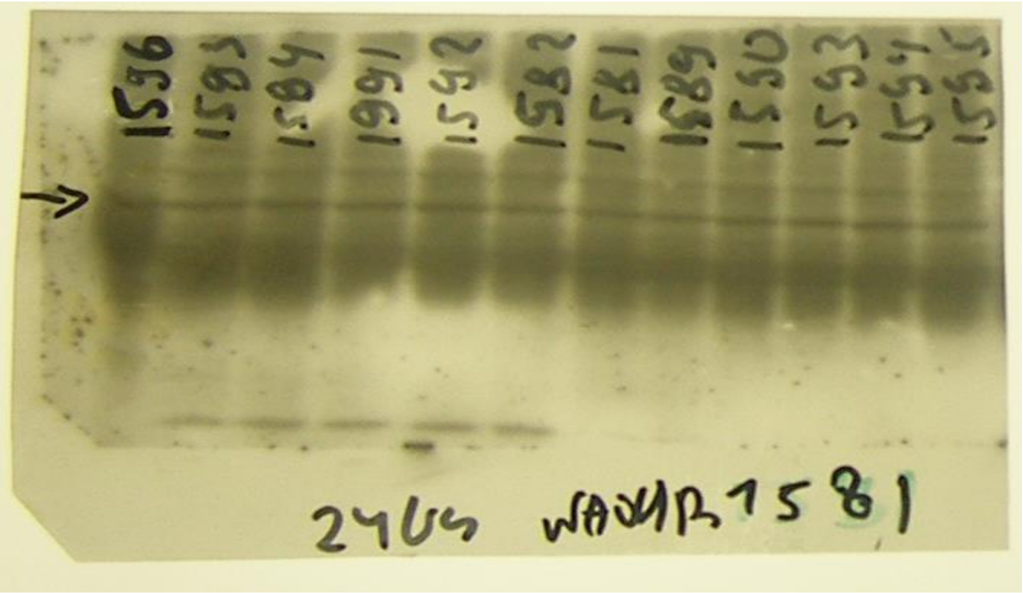

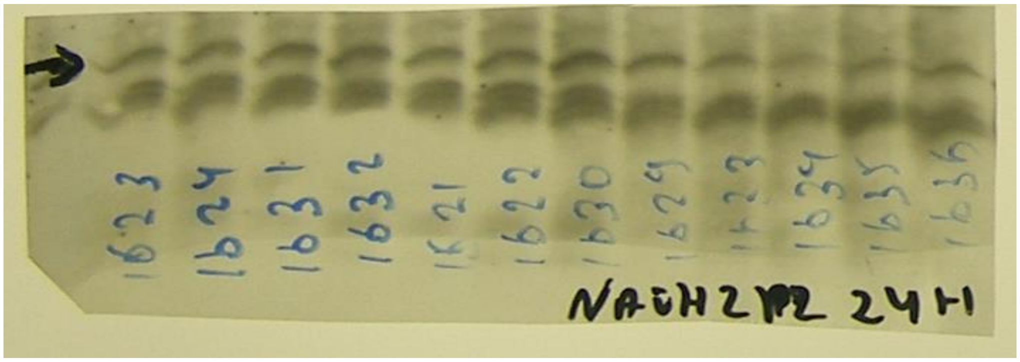

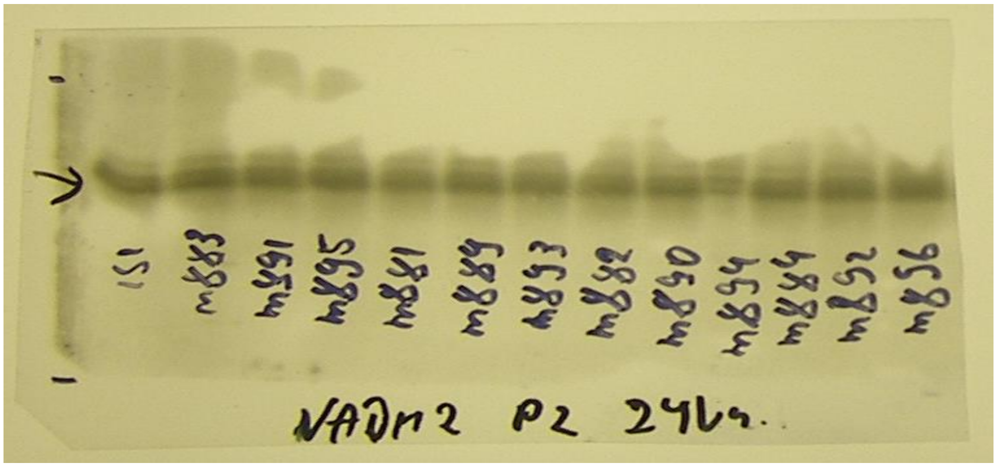

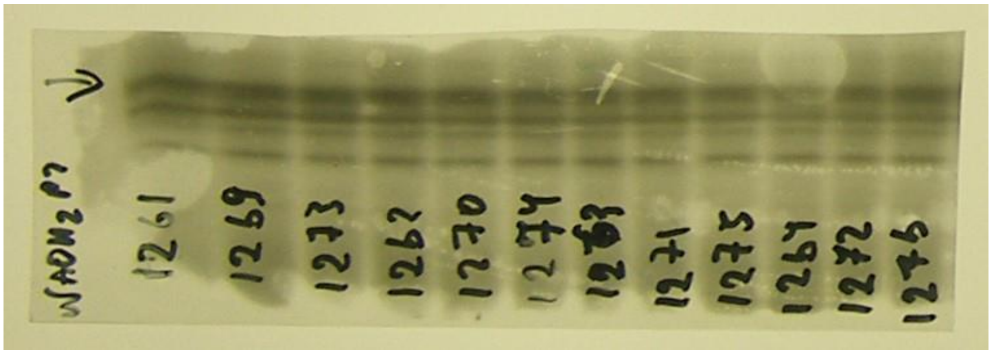

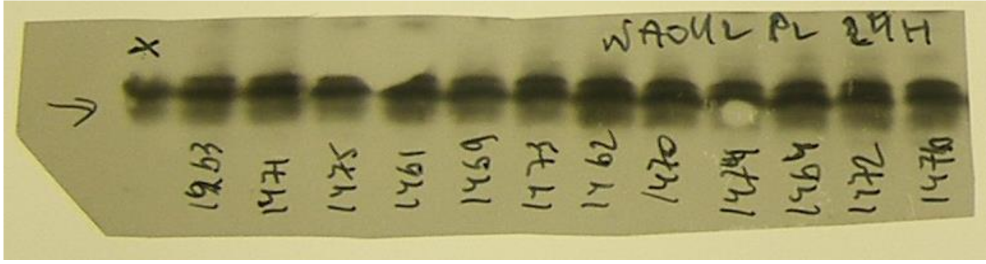

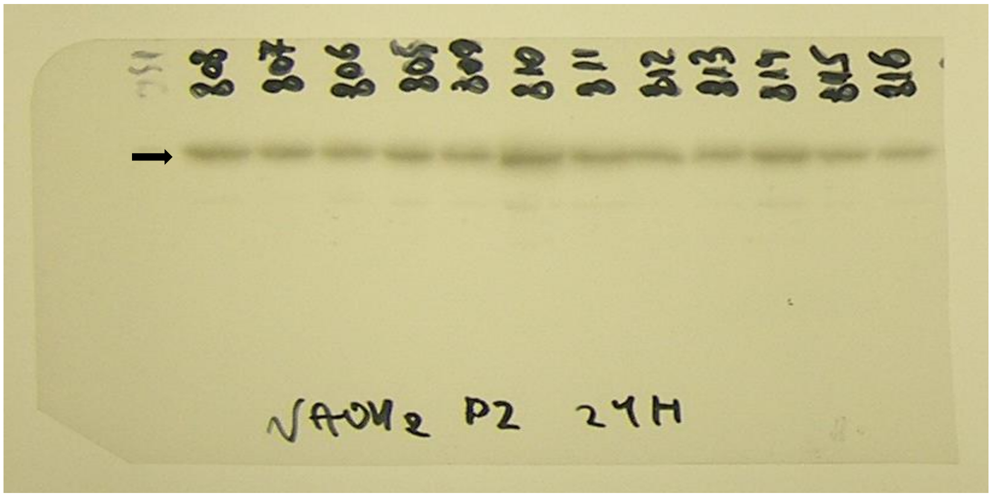

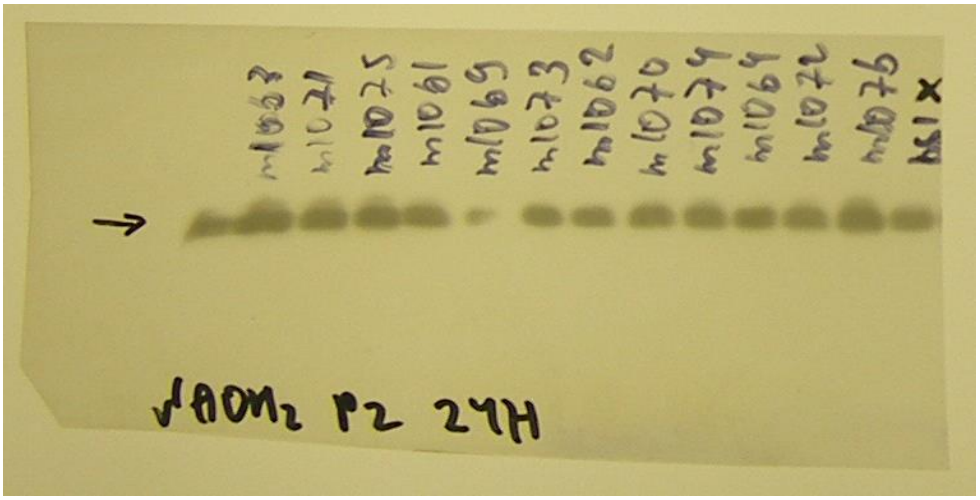
NAOH2 P2 24H

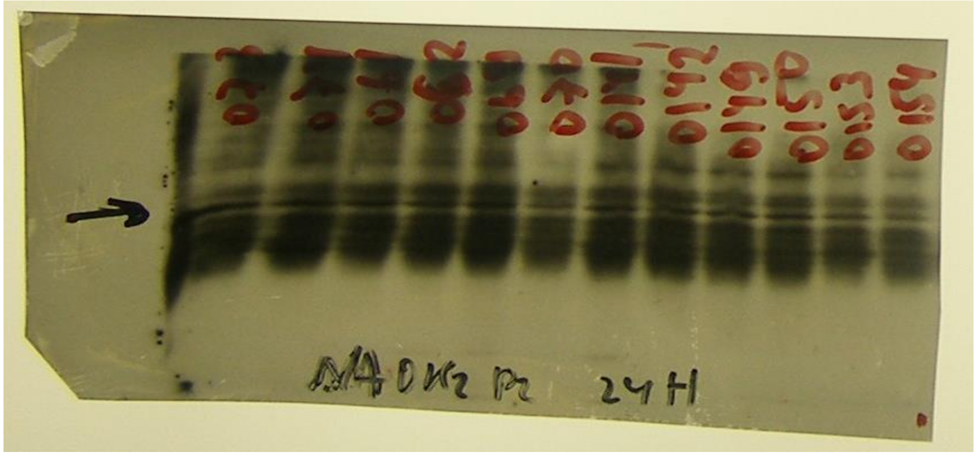
NAOH2 P2 24H

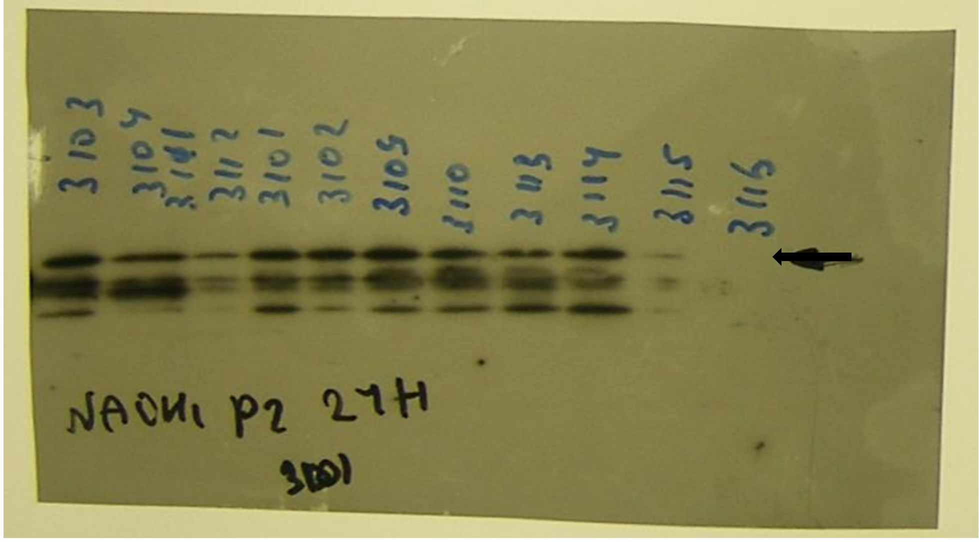
NAOH1 P2 24H
3001
