# Supplementary material for: Src-NADH dehydrogenase subunit 2 complex and recognition memory of imprinting in domestic chicks
Source: PLoS One. 2024 Jan 29;19(1):e0297166. doi: 10.1371/journal.pone.0297166 (PMC10824410; doi:10.1371/journal.pone.0297166)
Supplement: S1 File — (DOCX) [file pone.0297166.s015.docx]

Chick training and apparatus

Fertile eggs (Cobb 500) were obtained from Sabudara poultry,Tbilisi, Georgia. Twenty batches of eggs were incubated and hatched in darkness. The chicks were reared in isolation in darkness and at 22–28 h post-hatch trained for 1 h. Chicks from 9 batches were used in experiments in which measurements were made 1 h after the end of training; in the remaining eleven batches measurements were made 24 h after the end of training. In each batch, there were up to three trained chicks and a control chick from the same hatch. During training each chick was exposed in a running wheel (1 revolution = 94 cm) to a training stimulus (a cuboidal red box rotating about a vertical axis) for 1 h. The box contained a light surrounded by a red filter (Lee

Filters 106 Primary Red); the larger two sides of the box (18 × 18 cm) were translucent and vertical and the remaining sides (18 × 9 cm) were black. During training, the stimulus was turned on for 50 s and then off for 10 s each minute. The maternal call (70–75 dB) of a hen was played while the stimulus was on, a procedure that accelerates imprinting to a visual stimulus (Smith

and Bird, 1963). As a chick attempted to approach the training stimulus, it rotated the running wheel and revolutions of the wheel were counted to provide a measure of approach activity

(“training approach”). A preference test was performed without the maternal call 10 min after the end of 1 h training. During the test each chick in a running wheel was shown sequentially the training stimulus and an alternative stimulus that the chick had not previously seen, in the order training/alternative/alternative/training. Each period of exposure during the test lasted 4 min, making a total of 8 min for each stimulus. The alternative stimulus was a right circular

cylinder (height 18 cm and diameter 15 cm) with a translucent wall and vertical axis, rotating about this axis at 28 revolutions per minute. The cylinder contained a light surrounded by a blue

filter (Lee filters HT 118 Brilliant Blue). See Horn (1998) for illustrations of the training and alternative stimuli. A preference score (approach to training stimulus during test × 100/total

approach during test) measured the strength of imprinting (i.e., learning). A preference score of ∼50 indicates poor learning, whereas a score of ∼100 indicates strong learning. There are

individual differences in the preference scores of chicks after a fixed period of training. This variation was used to determine whether the amount of protein was correlated with preference score and, by subsequent analysis, whether a change in protein amount was attributable to learning that occurred during training

References

Smith, F. V., and Bird,M.W. The relative attraction for the domestic chick

of combinations of stimuli in different sensory modalities. Anim. Behav. 1963;11,

300–305. doi: 10.1016/S0003-3472(63)80115-3

Horn, G. Visual imprinting and the neural mechanisms of recognition

memory. Trends Neurosci. 1998; 21, 300305. doi: 10.1016/S0166-2236(97)

01219-8
